# Acoustically Activatable Drug‐Loaded Nanodroplets for Mechanochemical Therapy in Solid Tumors

**DOI:** 10.1002/advs.78010

**Published:** 2026-09-27

**Authors:** Tiran Bercovici, Mike Bismuth, Meir Goldsmith, Dan Peer, Tali Ilovitsh

**Affiliations:** ^1^ School of Biomedical Engineering Tel Aviv University Tel Aviv Israel; ^2^ Laboratory of Precision Nanomedicine The Shmunis School of Biomedicine and Cancer Research George S. Wise Faculty of Life Sciences Tel Aviv University Tel Aviv Israel; ^3^ Department of Materials Sciences and Engineering Iby and Aladar Fleischman Faculty of Engineering Tel Aviv University Tel Aviv Israel; ^4^ Cancer Biology Research Center Tel Aviv University Tel Aviv Israel; ^5^ Center For Nanoscience and Nanotechnology Tel Aviv University Tel Aviv Israel; ^6^ The Sagol School of Neuroscience Tel Aviv University Tel Aviv Israel

**Keywords:** acoustic droplet vaporization, cancer therapy, controlled drug release, focused ultrasound, mechanotherapy, microfluidic nanoassembly, nanodroplet

## Abstract

Stimulus‐responsive nanomedicines promise controlled therapy, yet most systems rely on passive delivery and lack precise, externally programmable activation while maintaining clinical compatibility. Here we engineer sub‐200 nm, perfluorocarbon (PFC)‐core nanodroplets (ND) that integrate efficient drug loading, physiological stability, and acoustically programmable activation within a nanoscale agent. These NDs are fabricated using microfluidic nanoassembly to achieve controlled size and composition, and are designed to encapsulate fluorinated payloads within the liquid core. Upon exposure to sequential dual‐frequency ultrasound (US), the NDs undergo acoustic droplet vaporization followed by low‐frequency cavitation, enabling spatially confined disruption and on‐demand payload release within clinically relevant acoustic limits. These properties are engineered to overcome physicochemical barriers in solid tumors, including dense extracellular matrix and restricted drug penetration. This approach achieves enhanced payload release and induces mechanochemical cytotoxicity in vitro. In vivo, NDs exhibit prolonged circulation and tumor accumulation, while US activation drives tissue fractionation, controls drug release, and increases subsequent nanoparticle uptake. When applied to a solid tumor model, this combined mechanochemical strategy improves tumor control and extends survival compared to either modality alone. These acoustically activatable NDs provide a versatile system for stimulus‐responsive, site‐targeted drug delivery and tumor disruption, with strong potential for clinical translation.

## Graphical abstract

Microfluidically fabricated drug‐loaded nanodroplets are sequentially activated by dual‐frequency ultrasound through acoustic droplet vaporization followed by bubble collapse, enabling spatially controlled drug release and mechanical tumor fractionation, resulting in synergistic mechanochemotherapy and prolonged survival.

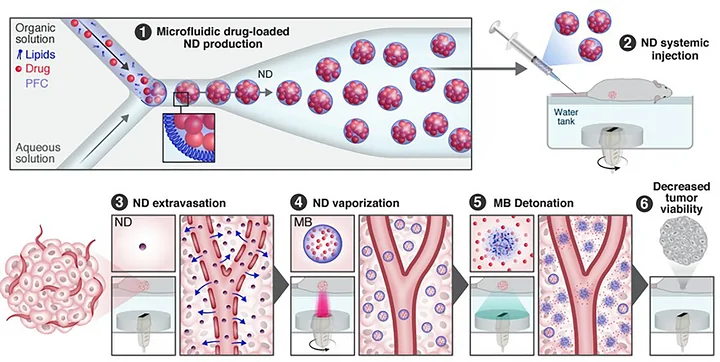

## 1 Introduction

Solid tumors remain therapeutically challenging due to their immunosuppressive microenvironment and pronounced physicochemical barriers, including dense extracellular matrix that restrict drug penetration and reduce therapeutic efficacy [1, 2, 3, 4, 5, 6, 7]. Effective cancer treatment requires a combination of strategies to reduce tumor burden and adjuvant therapies aimed at eliminating residual malignant cells. Current cancer treatment strategies include a combination of surgery, radiotherapy, and chemotherapy, with chemotherapy being one of the most widely used approaches [8, 9]. However, conventional chemotherapy often induces severe off‐target toxicity due to the nonspecific systemic distribution of cytotoxic drugs [10, 11]. To mitigate this limitation, nanocarrier‐based drug delivery systems have been developed to improve tumor localization and reduce systemic exposure [12, 13, 14, 15]. However, most clinically deployed nanomedicines rely on passive delivery mechanisms and lack user‐controlled, externally triggered drug release.

Ultrasound (US) is the most widely used medical imaging modality due to its safety, cost‐effectiveness, and noninvasive nature [16]. When a US beam is focused into a confined focal region, it can induce localized bioeffects that enable therapeutic action through thermal or mechanical mechanisms [17]. These effects can be exploited to trigger site‐specific uncaging of nanomedicine. Thermal approaches, such as temperature‐sensitive liposomes, exploit US‐induced hyperthermia to release encapsulated drugs [18]. While effective, these strategies often require prolonged sonication and are susceptible to heat diffusion, which can lead to unintended drug release in surrounding tissues [19]. Mechanical bioeffects provide an alternative activation mechanism, enabling localized cavitation and tissue disruption without bulk heating. However, existing US‐responsive particles do not simultaneously achieve high drug‐loading efficiency, physiological stability, and predictable acoustic activation [20, 21]. Microbubbles (MB), the most commonly used US contrast agents, consist of a gas core stabilized by a shell and typically measure 1–10 µm in diameter. Under US excitation, MBs act as cavitation nuclei [22, 23, 24], yet they are suboptimal drug carriers, as most drug‐loading strategies are confined to the shell [25]. Nanobubbles offer improved tumor penetration via the enhanced permeability and retention (EPR) effect [26], although their drug encapsulation capacity is even lower than that of MBs, due to their smaller size [20, 27]. Advanced liposomal systems enable controlled drug release but lack the mechanical bioeffects necessary for tumor debulking [18, 19].

Nanodroplets (ND), consisting of a liquid perfluorocarbon (PFC) core stabilized by a shell, were introduced to address some of these limitations [28, 29, 30]. Upon exposure to US, NDs can undergo acoustic droplet vaporization (ADV), transitioning from a liquid droplet into a gas MB [31]. The resulting bubble can serve as US imaging contrast and enhance mechanical effects such as cavitation and tissue disruption [20, 32]. Because NDs are smaller than MBs, they can circulate longer in the bloodstream, extravasate into tumors via the EPR effect, and then be activated on demand in the target tissue [30, 31, 33, 34]. Despite these advantages, current ND platforms for drug delivery face multiple challenges. First, many formulations suffer from limited size control and high polydispersity. Most reported NDs are fabricated using bulk or emulsion‐based methods such as sonication, homogenization, thin‐film hydration, or MB condensation. These approaches provide limited control over nucleation and interfacial assembly, resulting in broad size distributions, batch‐to‐batch variability, and often increased particle diameters [35, 36, 37]. Recent work with droplet‐based microfluidics has shown that it is possible to generate highly uniform droplets with tunable size and composition, opening the door to more reproducible and controllable ND formulations [36, 38, 39, 40]. Second, ND vaporization behavior is strongly dependent on multiple parameters, including the PFC core, ND diameter, US frequency, and applied peak negative pressure (PNP) [41, 42]. Condensation‐based techniques commonly employ perfluorobutane (C_4_F_10_), which has a low boiling point (−1.7°C) and is gaseous at room temperature. While such formulations have demonstrated efficacy for US mechanotherapy [43], they can be less stable under physiological conditions and prone to spontaneous vaporization [28]. To improve in vivo stability, higher boiling point PFCs such as perfluoropentane (C_5_F_12_; boiling point ∼29°C) and perfluorohexane (C_6_F_14_; boiling point ∼56°C) are therefore often used [28, 44]. However, this increased stability comes at the cost of higher vaporization thresholds [28, 44]. Third, while we have shown that insonating MBs at low frequencies enables noninvasive US surgery by significantly reducing the PNP required to induce cavitation [24, 45], ND vaporization typically requires PNPs in the MPa range [44, 46, 47, 48]. At low frequencies, these pressures often exceed FDA‐approved safety limits for diagnostic imaging (mechanical index < 1.9). This challenge is further amplified for smaller NDs and higher boiling point PFCs, which require even higher PNPs for vaporization [44, 49]. Finally, efficient drug loading into the PFC core remains a major challenge. Most existing approaches rely on shell loading or double‐emulsion strategies, which either limit payload capacity or increase droplet diameter, thereby compromising tumor penetration and systemic delivery [20, 50].

**FIGURE 1:**
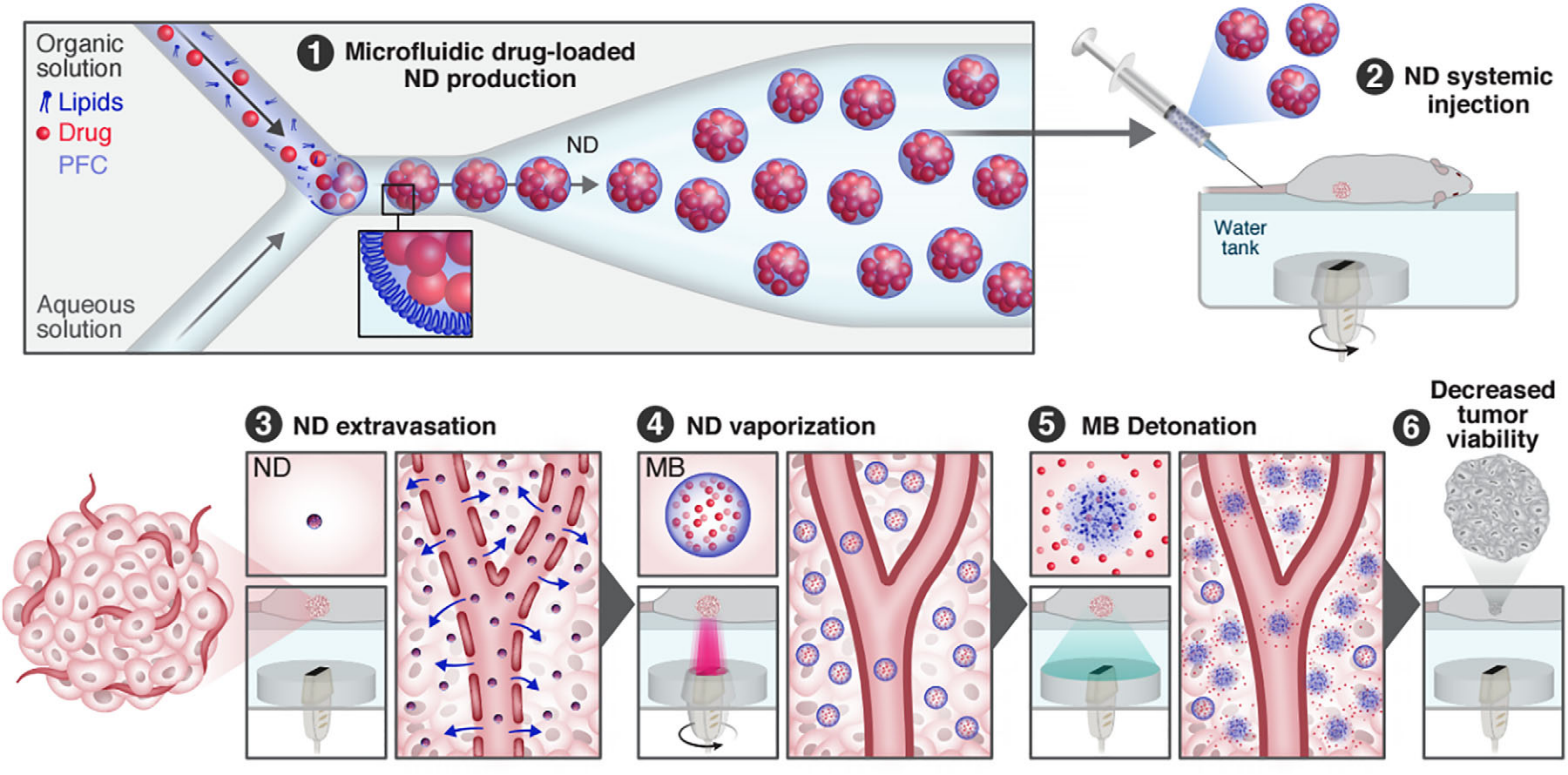
Schematic illustration of drug‐encapsulated ND synthesis and dual‐frequency mode US mechanotherapy. First, drug‐loaded NDs are fabricated via nanoassembly microfluidic synthesis. Following systemic ND injection, a rotating imaging transducer initiates ADV to vaporize NDs into MBs. Subsequent low‐frequency therapeutic US drives high‐amplitude oscillations of the vaporized NDs, inducing volumetric tumor tissue fractionation and drug uncaging for combined mechanotherapy and localized drug delivery.

Here we introduce an acoustically activatable ND platform designed to enable controlled droplet fabrication, efficient drug loading into the core, and enhanced US‐triggered therapeutic activation within a single nanoscale agent. We employ microfluidic nanoassembly to achieve precise control over ND size (<200 nm), composition, and concentration, enabling reproducible vaporization behavior and enhanced tumor extravasation. To ensure physiological stability while preserving acoustic responsiveness, higher‐order PFC cores are incorporated. Efficient drug loading is achieved through rational selection of fluorinated therapeutic molecules that exhibit physicochemical compatibility with the PFC phase, enabling dissolution within the droplet core without increasing ND size. Fluorination has been shown to enhance metabolic stability and pharmacokinetic performance, with many fluorinated drugs already approved or in clinical use [51, 52, 53, 54, 55, 56, 57, 58]. Using this strategy, we develop three classes of NDs, including blank NDs, fluorophore‐loaded NDs incorporating BODIPY dyes for mechanistic characterization, and chemotherapy‐loaded NDs using 5‐fluorouracil (5‐FU) as a model cytotoxic agent. To enable treatment within FDA safety limits, we implement a dual‐frequency US strategy (Figure 1). A rotating MHz‐frequency imaging transducer first induces controlled ADV within FDA mechanical index (MI) constraints, vaporizing NDs into MBs. Subsequently, a low‐frequency therapeutic transducer drives vaporized ND collapse, generating potent mechanical fractionation and on‐demand payload release at the target site. This sequential activation approach reduces the required PNP for therapeutic bioeffects while preserving spatial precision and safety. Together, this integrated design establishes a generalizable framework for low‐energy, mechanically active US nanomedicine capable of simultaneous tumor debulking and controlled drug uncaging, thereby advancing the therapeutic potential of acoustically responsive nanocarriers for solid tumor treatment.

## 2 Results

### 2.1 Ultrasound‐Responsive ND Preparation, Size Distribution, and Stability

**FIGURE 2:**
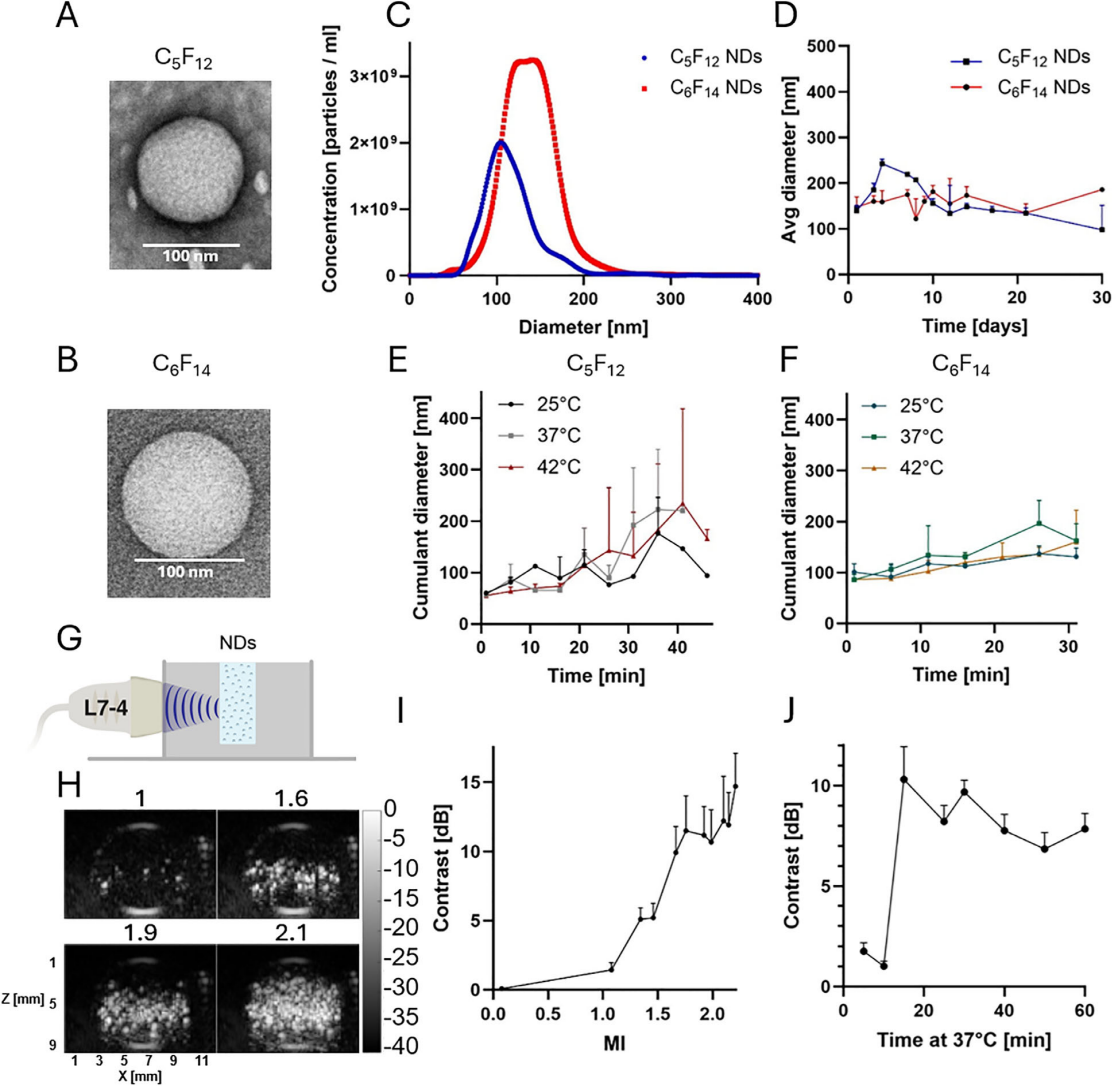
ND physicochemical and acoustic response characterization. (A, B) TEM images showing the spherical morphology of (A) C_5_F_12_, (B) C_6_F_14 _NDs. Scale bars: 100 nm. (C) NTA size distribution profiles comparing the hydrodynamic diameter and particle concentration of C_5_F_12_ (blue) vs. C_6_F_14_ (red) NDs. (D) Long‐term colloidal stability of C_5_F_12_ and C_6_F_14_ NDs stored at 4°C for 30 days. Measurements were performed at room temperature using DLS. Data represent mean diameter ± SD (*n* = 3). (E, F) Thermal stability analysis via DLS, monitoring hydrodynamic size variations at ambient (25°C), physiological (37°C), and hyperthermic (42°C) temperatures for (E) C_5_F_12_ and (F) C_6_F_14_ NDs. (G) Experimental setup for ADV characterization of C_6_F_14_ NDs employing an L7‐4 linear array transducer (5.5 MHz). C_6_F_14_ NDs (diluted 1:100 in degassed PBS) were injected into a cylindrical inclusion within an agarose tissue‐mimicking phantom to assess ADV thresholds. (H)  B‐mode US images showing pressure‐dependent ADV at 37°C, from no detectable vaporization at MI 1.0 to robust vaporization at MI 1.9–2.1. (I) Quantification of contrast enhancement as a function of MI, showing an ADV threshold at ∼MI 1.6. (J) Temporal stability of vaporization‐induced contrast at 37°C and MI 1.9, with repeated insonation at the indicated time points (black dots).

The first step was to optimize the microfluidic preparation method and the ND core composition. To this end, US‐responsive NDs were formulated using two PFC cores, C_5_F_12_ and C_6_F_14_, via microfluidic mixing on two platforms: a glass herringbone microfluidic chip and the NanoAssemblr system (Figure 2). The NanoAssemblr system operates at higher total flow rates (TFR), typically 4–24 mL/min, whereas the glass herringbone chip is generally limited to lower flow rates (0.1–5 mL/min), making the NanoAssemblr more suitable for scaled‐up production and formation of smaller NDs. NDs were generated at a fixed aqueous‐to‐organic flow rate ratio (FRR) of 3:1, while varying the TFR to modulate particle size (100–2750 µL/min for the Herringbone Mixer Glass Chip and 12 mL/min for the NanoAssemblr device). This strategy enabled systematic tuning of ND size and concentration as a function of TFR. Nanoparticle Tracking Analysis (NTA) was used to quantify ND size distributions and concentrations (Table 1).

**TABLE 1:** Effect of microfluidic preparation conditions on ND size and concentration measured by NTA.

	TFR [mL/min]		C_5_F_12_ ND	C_6_F_14_ ND
Herringbone microfluidic chip	0.1	Diameter [nm]	242.9 ± 9.3	—
		Concentration [particles/mL]	1.07 × 10^10^ ± 2.67 × 10^8^	—
	0.3	Diameter [nm]	238.8 ± 14.7	—
		Concentration [particles/mL]	1.09 × 10^10^ ± 8.18 × 10^8^	—
	0.5	Diameter [nm]	226.8 ± 1.4	230.0 ± 3.6
		Concentration [particles/mL]	6.46 × 10^10^ ± 1.56 × 10^9^	1.91 × 10^10^ ± 6.37 × 10^9^
	2.0	Diameter [nm]	191.9 ± 2.1	210.3 ± 3.2
		Concentration [particles/mL]	4.61 × 10^10^ ± 2.60 × 10^9^	3.71 × 10^10^ ± 6.96 × 10^8^
	2.75	Diameter [nm]	156.9 ± 2.3	185.1 ± 4.5
		Concentration [particles/mL]	9.61 × 10^9^ ± 3.01 × 10^8^	1.37 × 10^10^ ± 1.93 × 10^8^
Nano Assemblr	12.0	Diameter [nm]	117.4 ± 2.2	139.5 ± 0.3
		Concentration [particles/mL]	1.25 × 10^11^ ± 6.44 × 10^9^	2.52 × 10^11^ ± 4.90 × 10^9^

At a high TFR of 12 mL/min on the NanoAssemblr platform with FRR  =  3:1, C_5_F_12_ and C_6_F_14_ NDs exhibited mean diameters of 117.4  ±  2.2 nm and 139.5  ±  0.3 nm, respectively, with corresponding particle concentrations of 1.25  ×  10^11^  ±  6.44  ×  10^9^ and 2.52  ×  10^11^  ±  4.90  ×  10^9^ particles/mL (Figure 2c). Lower TFR (2.00–2.75 mL/min) using the Herringbone Mixer Glass Chip generated larger NDs (by ∼40%–50% compared to NanoAssemblr at 12 mL/min) and lower particle concentrations across both PFC cores (Table 1). Under identical TFR conditions, C_5_F_12_ consistently produced smaller NDs than C_6_F_14_ (by ∼25%–35% across TFRs tested). TEM and E‐SEM imaging confirmed spherical morphology for both NanoAssemblr‐generated formulations (TEM: Figure 2a,b and E‐SEM: Figure S1a,b). Zeta potential measurements revealed superior colloidal stability for C_6_F_14_ NDs (−29.8 ± 3.7 mV) compared to C_5_F_12_ NDs (−20.6 ± 3.0 mV), attributable to differences in shell–core interactions and surface charge density. Given the smaller size achievable with the NanoAssemblr, all subsequent in vitro and in vivo experiments were conducted using NanoAssemblr‐fabricated NDs.

Next, stability tests of the two formulations were carried out over 30 days using dynamic light scattering (Figure 2d). C_6_F_14_ NDs exhibited superior stability (Figure 2d), with average size deviating by up to + 26% from baseline (147.2 ± 18.1 nm at day 1). In contrast, C_5_F_12_ NDs were less stable (Figure 2d), with size increasing by up to + 75% by day 5 and decreasing by −30% by day 30 relative to day 1. This can be attributed to their lower boiling point and greater susceptibility to Ostwald ripening or spontaneous phase transitions under storage conditions.

Thermal stability was additionally assessed at 25°C, 37°C, and 42°C, mimicking the physiological temperature range for up to 30 min (Figure 2e,f). C_6_F_14_ NDs remained stable, whereas C_5_F_12_ NDs exhibited non‐monotonic size fluctuations, consistent with their lower boiling point.

Long‐term assessments after 60 days (Figure S1c,d) confirmed the superior stability of C_6_F_14_ NDs across all temperatures. Based on their improved storage and thermal stability, C_6_F_14_ NDs were selected for all subsequent experiments.

### 2.2 Acoustic Activation and Vaporization Behavior of NDs

NDs were next evaluated for acoustic responsiveness and vaporization thresholds. At 25°C, neither C_5_F_12_ nor C_6_F_14_ NDs exhibited detectable ADV up to MI 2.1, whereas increasing temperature reduced the vaporization threshold (Figure S2). At 37°C, C_6_F_14_ ND vaporization was pressure‐dependent, with contrast increasing from 5 ± 0.8 dB at MI 1.5 to 14 ± 2.4 dB at MI 2.1 and robust ADV observed at MI ≥ 1.9 (Figure 2g–i and Figure S3). These results define an activation window between MI 1.58 and 1.9 at 37°C. Overall, C_6_F_14_ NDs exhibited a higher vaporization threshold than C_5_F_12_ NDs, supporting greater stability under physiological conditions.

Microfluidic flow rate further influenced ND stability and acoustic responsiveness. C_6_F_14_ NDs fabricated at lower TFRs (1.0 and 2.75 mL/min) exhibited partial ADV at low MI, whereas NDs fabricated at 12 mL/min remained stable at MI 0.07 and underwent robust ADV at MI 1.9 (Figure S4). Together with their smaller size (Table 1), these findings supported selection of the 12 mL/min formulation for subsequent experiments.

The stability of ADV‐generated MBs was further evaluated during repeated US exposure at 37°C and MI 1.9. Contrast increased rapidly during the first 15 min (10.3 ± 1.6 dB) and subsequently reached a stable plateau, indicating sustained post‐vaporization stability (Figure 2j). Finally, low‐frequency US applied after ADV eliminated the vaporization‐induced contrast, returning the signal to baseline and demonstrating collapse of the ADV‐generated MBs (Figure S5).

### 2.3 Drug Loading and ND Functionalization

**FIGURE 3:**
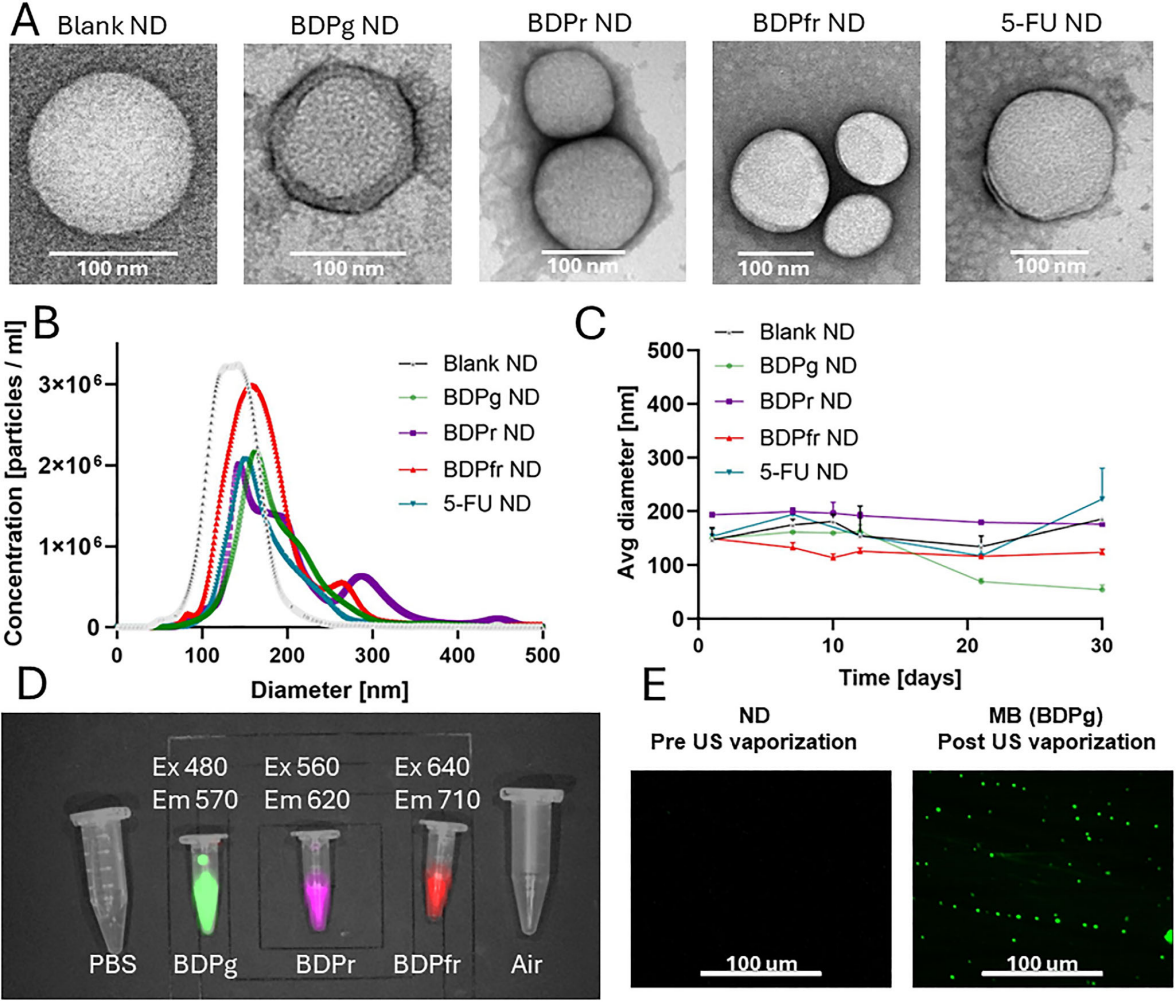
Morphology, stability, and optical properties of BODIPY‐encapsulated NDs. (A)  Transmission electron microscopy of blank NDs, fluorophores‐loaded ND (BDPg, BDPr, BDPfr), and chemotherapy (5‐FU) ND. All formulations maintain a spherical structure post‐encapsulation. Scale bar: 100 nm. (B) Comparative NTA profiles displaying the size distribution and particle concentration for blank, dye‐loaded, and drug‐loaded NDs. (C) Long‐term stability analysis tracking the average hydrodynamic diameter of blank and encapsulated NDs over a 30‐day storage period. (D) IVIS fluorescence imaging of Eppendorf tubes containing PBS (control), air (control), and the three BODIPY‐loaded ND variants. The panel highlights successful spectral separation and distinct fluorescence emission for BDPg (Ex 480 nm/Em 570 nm), BDPr (Ex 560 nm/Em 620 nm), and BDPfr (Ex 640 nm/Em 710 nm) NDs. (E) Fluorescence microscopy of BDPg ND before and after US exposure. The Pre‐US state shows no detectable fluorescence structures, while the Post‐US state reveals the formation of green‐fluorescent MBs. Scale bar: 100 µm.

After confirming the acoustic activity of the particles, we next functionalized the C_6_F_14_ NDs by encapsulating fluorescent molecules and chemotherapeutic drugs. A summary of the physical properties of the PFC core (C_5_F_12_ and C_6_F_14_) and other materials (chemotherapy and fluorescent molecules) used for the formation of the NDs is provided in Table S1. Fluorescently labeled NDs were used to quantify in vivo biodistribution and intratumoral penetration in a breast cancer model, whereas drug‐loaded NDs enabled combined mechanotherapy and chemotherapy. Fluorescently labeled NDs utilized BODIPY derivatives, including BODIPY variants spanning green (BODIPY 493/503; BDPg), red (BDP TR‐X‐NHS ester; BDPr), and far‐red (BDP 630/650‐X‐NHS ester; BDPfr) emission wavelengths to enable multichannel fluorescence detection and mechanistic studies. This fluorophore class provides high quantum yield and photostability [59, 60], with fluorinated side groups enabling direct C_6_F_14_ core dissolution and stable encapsulation without size increase. Chemotherapy NDs encapsulated 5‐fluorouracil (5‐FU). These materials were selected for their fluorinated structures, low aqueous solubility, and established utility in imaging/chemotherapeutic applications. Size, stability, zeta potential, and phase‐change behavior were characterized across formulations. All encapsulated NDs exhibited comparable size distributions via NTA (Table 2). Across BODIPY, 5‐FU‐loaded, and blank formulations, NDs showed average diameters of ∼140–160 nm, concentrations of 1.2–2.5 × 10^11^ particles/mL (Figure 3b,c), and zeta potentials between −21 and −36 mV (blank NDs ∼−30 mV), indicating robust colloidal stability. TEM confirmed well‐defined core–shell nanostructures (Figure 3a,b).

**TABLE 2:** Size distribution, concentration, and zeta potential of blank, BODIPY, and 5‐FU‐loaded ND.

ND type	Average size [nm]	Concentration [particles/mL] × 10^11^	Zeta potential [mV]
Blank ND	139.5 ± 0.3	2.52 ± 0.05	−29.80 ± 3.70
BDPg ND	162.3 ± 58.6	1.34 ± 0.11	−21.19 ± 0.96
BDPr ND	143.3 ± 79.0	1.53 ± 0.02	−21.48 ± 4.54
BDPfr ND	158.5 ± 60.7	2.11 ± 0.03	−36.33 ± 1.31
5‐FU ND	151.0 ± 45.5	1.18 ± 0.03	−23.07 ± 0.33

BODIPY and 5‐FU‐loaded NDs exhibited distinct 30‐day stability profiles (Figure 3c) under refrigerated storage (4°C; measurements at 25°C). BDPfr NDs (initial 193.5 ± 1.4 nm) demonstrated long‐term stability, maintaining diameters within ∼8% of initial size through day 30. 5‐FU NDs (initial 154.0 ± 13.7 nm) showed moderate stability with fluctuations up to ± 45% relative to baseline over 30 days. In contrast, BDPg NDs (initial 149.4 ± 3.4 nm) were less stable, exhibiting a progressive size reduction of ∼65% by day 30.

Multispectral IVIS analysis characterized the excitation and emission properties for each BODIPY‐loaded ND formulation. BDPg NDs exhibited peak fluorescence at 480/570 nm, BDPr NDs at 560/620 nm, and BDPfr NDs at 640/710 nm (Figure 3d). These optimized spectral pairs provided maximal signal intensity for each type while maintaining separation between formulations, and specificity was further validated by the absence of signal in control samples (PBS and air blanks). Consistent with efficient core encapsulation, fluorescence microscopy demonstrated minimal residual BODIPY in the supernatant after dialysis (Figure 3e).

### 2.4 Confocal Microscopy of Microdroplets

To visually confirm payload localization in the core, we sought to resolve the core and shell compartments directly. Owing to the small size of the NDs, confocal microscopy, with a lateral resolution of >250 nm, cannot reliably resolve the core–shell structure. Therefore, to enable direct visualization, we fabricated micron‐sized droplets using the microfluidic platform. These microdroplets were co‐loaded with BDPg (green) in the core and DiI (red) in the lipid shell using the Herringbone chip with aqueous‐to‐organic phase flow rates of 150:50 µL/min. Confocal microscopy images (Figure S6a–c) demonstrate BODIPY encapsulation within the PFC core, with green fluorescence originating from the droplet interior and DiI (red) localized to the shell.

### 2.5 ADV, Encapsulation Efficiency and Cavitation‐Triggered Drug Release

**FIGURE 4:**
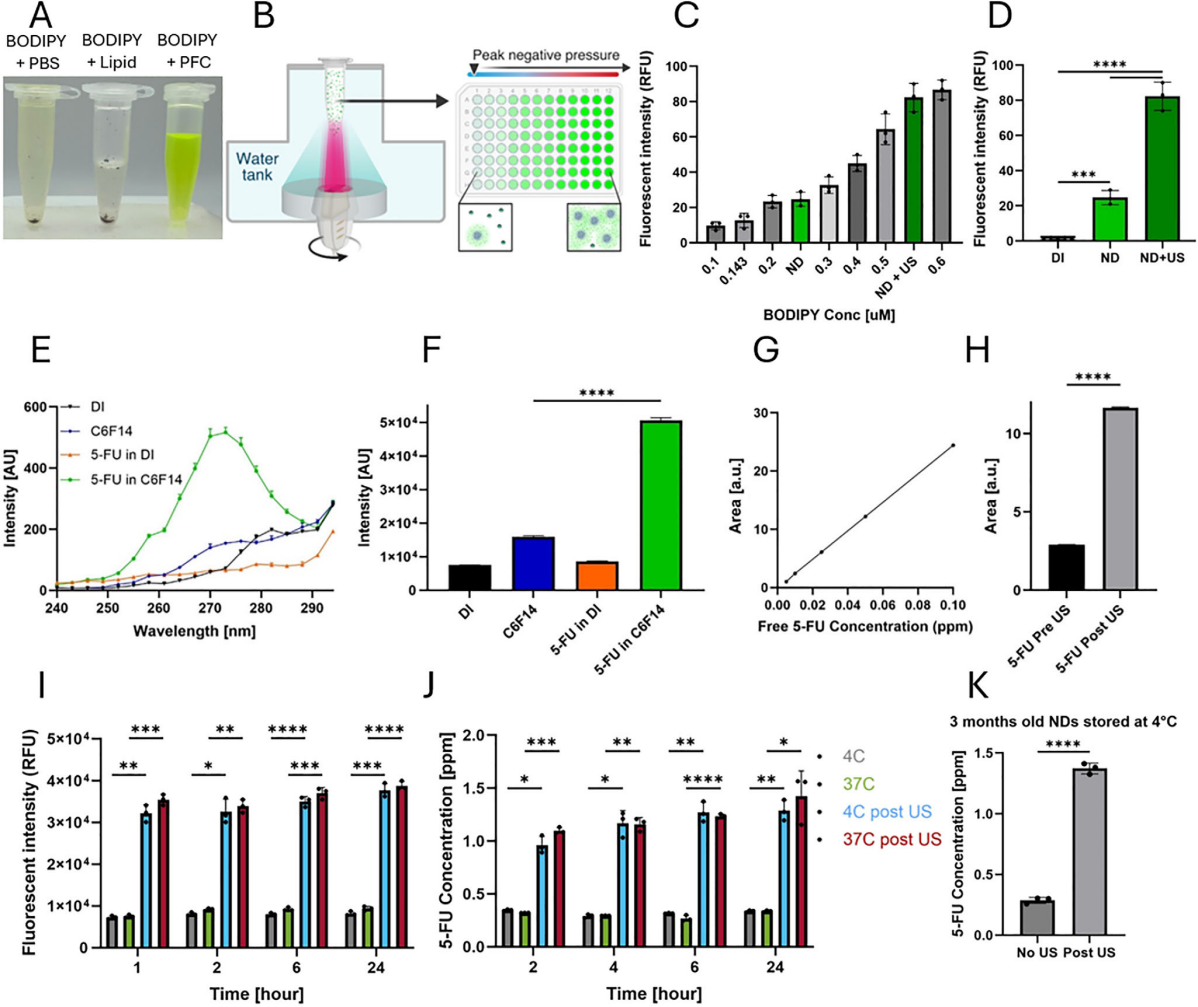
US‐triggered payload release kinetics and validation of core‐loading solubility. (A) Solubility comparison of BODIPY in different solvents. Left and Middle: BODIPY shows limited solubility in PBS or lipid solution, with visible precipitate at the bottom of the tube. Right: BODIPY in PFC (C_6_F_14_) demonstrates dissolution, yielding a homogeneous green‐yellow solution. (B) Schematic illustration of the in vitro release experimental setup. Payload release was triggered using a simultaneous dual‐frequency US system (3.5 MHz imaging transducer, MI 1.67; 105 kHz therapy transducer, MI 0.84). Release efficiency was quantified by analyzing the fluorescence/absorbance of the supernatant fraction collected before and after US exposure. (C) Quantification of BDPg release showing the calibration curve (fluorescence intensity vs. concentration, R^2^ = 0.979) overlaid with supernatant measurements. A ∼4‐fold increase in fluorescence intensity is observed in the post‐US supernatant compared to pre‐US controls. (D) Comparative fluorescence analysis of top supernatant fractions from DI water (background), intact NDs (passive leakage), and US‐activated NDs (active release). (E) Spectroscopic characterization of 5‐FU solubility. Representative excitation spectra (Em = 310 nm) of 5‐FU dissolved in C_6_F_14_ vs. DI water, revealing a characteristic intensity peak at ∼270 nm specific to the fluorinated phase. (F) Quantification of 5‐FU fluorescence intensity (Ex = 265 ± 20 nm, Em = 310 ± 20 nm) in C_6_F_14_ and DI water compared to pure solvent controls. The significant signal enhancement confirms the successful dissolution and core‐loading of 5‐FU within the C_6_F_14_ matrix. (G) HPLC calibration curve correlating peak area with free 5‐FU concentration, used to calculate the release concentrations in panel H (R^2^ = 0.99). (H) HPLC quantification of 5‐FU release efficiency at 25°C, showing a significant increase in the integrated peak area (a.u.) for post‐US samples compared to pre‐US baselines (^****^ *p* < 0.0001). (I) Release profile of BDPg‐loaded NDs following US activation using the dual‐transducer system at 4°C and 37°C over 24 h. Passive release remained near baseline levels, whereas US exposure resulted in a pronounced increase in fluorescence intensity corresponding to an approximately 4‐ to 5‐fold enhancement in payload release. (J) Release profile of 5‐FU‐loaded NDs following US activation using the dual‐transducer system at 4°C and 37°C over 24 h. Passive release profile of 5‐FU‐loaded NDs at 4°C and 37°C showing minimal drug leakage, with supernatant concentrations remaining low and stable. US treatment significantly increased the supernatant 5‐FU concentration. (K) Release profile of 5‐FU NDs following US activation using the dual‐transducer system after storage at 4°C for 3 months. US treatment significantly increased the supernatant 5‐FU concentration. Data represent mean ± SD (*n* = 3). Statistical significance was calculated using one‐way ANOVA with Tukey's multiple comparison test, or (for I and J) two‐way ANOVA with Tukey's multiple comparison test (^*^ *p* < 0.05, ^**^ *p* < 0.01, ^***^ *p* < 0.001, ^****^ *p* < 0.0001).

Next, we sought to evaluate the ability of the NDs to achieve controlled release of the fluorescent payload. First, we assessed the solubility of free BODIPY (BDPg) in different solvents (Figure 4a), demonstrating that BODIPY remained undissolved in PBS solution or Lipid solution, but readily dissolved in C_6_F_14_ PFC solution, forming a homogeneous green‐yellow solution.

To determine whether BDPg and 5‐FU are molecularly dissolved in the PFC phase or instead form aggregates within the PFC core, DLS measurements were performed on pure Perfluorohexane (PFH, C_6_F_14_), BDPg in PFH (0.1 mg/mL), and 5‐FU in PFH (0.1 mg/mL). All samples exhibited a single, narrow population with hydrodynamic diameters in the sub‐nanometer range (Figure S7a,b): PFH only (0.95 ± 0.08 nm), BDPg in PFH (0.96 ± 0.04 nm), and 5‐FU in PFH (0.98 ± 0.03 nm). No larger populations or secondary peaks were detected (Figure S7a), indicating that both BDPg and 5‐FU are molecularly dissolved in the PFC phase.

Based on this solubility profile, we formulated BODIPY‐loaded NDs by dissolving the fluorophore in C_6_F_14_ and encapsulating the solution within a lipid shell. Then, we verified that the ADV capability was preserved. Upon US activation (L7‐4 transducer, 5.5 MHz, MI 1.9) of BODIPY NDs , ADV of the C_6_F_14_ core generated fluorescent MBs, visualized by microscopy (Figure 3e, pre/post‐vaporization).

Pre‐activation NDs (<200 nm) were undetectable at 20× magnification (100 µm scale bar), whereas post‐ADV MBs exhibited pronounced fluorescence enhancement attributable to ADV‐induced volume expansion. Similar results were obtained with red BODIPY NDs with fluorescent MB formation post‐activation (Figure S8a,b).

To determine encapsulation efficiency, BDPg NDs and 5‐FU NDs were used. The unencapsulated free payload was separated and measured in the supernatant prior to treatment via UV–vis spectrophotometry (for BDPg) or HPLC (for 5‐FU), while the total payload was quantified after dissolving the lipid shell matrix with acetonitrile to ensure complete cargo release.

For BDPg NDs, the fluorescence intensity of the whole solution (after acetonitrile treatment) was 47,157.333 ± 2,803.820 a.u., compared to 7,5‐179.333 ± 248.737 a.u. for the supernatant, yielding an %EE of 84.8 ± 4.6% (Figure S9a). For 5‐FU NDs, HPLC measurements based on the calibration curve (Figure S9b) showed whole solution values of 1.861 ± 0.095 ppm vs. 0.245 ± 0.003 ppm for the supernatant, corresponding to an encapsulation efficiency of 86.84% ± 0.69% (Figure S9c).

### 2.6 US‐Triggered Payload Release at Room Temperature

To evaluate US‐triggered release, a two‐step activation‐detonation process was applied using a US‐guided focused US system at room temperature. First, ND vaporization was induced using a rotating imaging array operating at a center frequency of 3.5 MHz and MI = 1.67 to enable volumetric ND activation. Subsequently, collapse of the vaporized NDs was driven using a therapeutic transducer operating at a center frequency of 105 kHz and MI = 0.84 (Figure 4b). Applying this sequence to BDPg NDs triggered substantial payload release, quantified by UV–vis spectrophotometry. Due to the high density of C_6_F_14_ (1.68 $\frac{g}{mL}$) relative to the aqueous medium, NDs sedimented, enabling supernatant analysis of free fluorophore. Calibration against BDPg standards (0.1–0.6 µm, Figure 4c) revealed a 2.8‐fold fluorescence increase post‐treatment (0.61 ± 0.11 µm supernatant vs. 0.22 ± 0.08 µm pre‐treatment, Figure 4d), confirming efficient ADV and cavitation‐triggered release with this dual‐frequency paradigm. After confirming the ability of the ND to release fluorescent molecules, we next evaluated their capacity to encapsulate chemotherapeutic drugs. First, we evaluated the solubility of 5‐FU in C_6_F_14_ compared to DI water using UV–vis spectrophotometry. Full‐spectrum scanning (λ_ex_ 240–295 nm, λ_em_ 310 ± 5 nm) revealed a distinct absorbance profile for 5‐FU in C_6_F_14_, characterized by a sharp intensity peak around 270 nm, significantly exceeding the signal observed in DI water (Figure 4e). Quantitative analysis was subsequently performed using the optimized parameters (λ_ex_ 265 ± 20 nm, λ_em_ 310 ± 20 nm), where 5‐FU dissolved in C_6_F_14_ exhibited a ∼6‐fold increase in fluorescence intensity compared to 5‐FU in DI water (Figure 4f). Control measurements of neat solvents showed baseline intensities of 15,910 ± 384 a.u. for C_6_F_14_ and 7,510 ± 79 a.u. for DI water. When corrected for solvent background, the net signal for 5‐FU in C_6_F_14_ (34,758 a.u.) was 31‐fold higher than in DI water (1,106 a.u.), confirming the superior solubility and retention of fluorinated 5‐FU molecules within the PFC phase.

US‐triggered 5‐FU release experiments at 25°C were then performed as described for the BODIPY‐loaded NDs. The released drug was quantified by HPLC using a calibration curve generated from free 5‐FU (0.005–0.1 ppm, R^2^  =  0.9861, Figure 4g). For analysis, 10 µL of supernatant was collected from the top of each 1 mL sample before and after US application, diluted 100‐fold, filtered, and processed for HPLC. These measurements confirmed an increase in 5‐FU concentration post‐sonication. At a flow rate of 0.8  mL/min with a 50 µL injection volume, the chromatographic peak area increased from 2.91 to 11.65 (Figure 4h), representing a 4.0‐fold increase. This translated to original solution concentrations rising from 1.2 ppm (pre‐US) to 4.8 ppm (post‐US), after correcting for dilution.

### 2.7 Thermal Stability and Controlled Payload Release From ND

Following the room‐temperature release presented above, we next evaluated how thermal conditioning impacts baseline payload retention vs. US‐triggered release yield over 24 h. To distinguish passive baseline leakage from US‐triggered release across storage (4°C) and physiological (37°C) conditions, time‐resolved payload retention profiles were quantified for both BDPg‐ and 5‐FU NDs across incubation periods extending up to 24 h, evaluated with and without exposure to our dual‐frequency US system using the same parameters as the RT experiments.

For BDPg NDs (Figure 4i), supernatant levels remained minimal at both 4°C and 37°C over 24 h, with fluorescence intensities staying close to baseline. In contrast, US‐triggered release induced a rapid increase in fluorescence intensity at both temperatures, reaching 4‐ to 5‐fold higher fluorescence compared with passive controls (^**^ *p* < 0.01, ^***^ *p* < 0.001, ^****^ *p* < 0.0001). For 5‐FU NDs (Figure 4j), passive release remained minimal at both 4°C and 37°C throughout the 24 h study. At 4°C, the supernatant concentration did not change significantly after 24 h (0.347 vs. 0.336 ppm after dilution 0.3:1, ∼3% decrease, not significant), and also not at 37°C (0.321 vs. 0.336 ppm after dilution 0.3:1, ∼5% increase, not significant), demonstrating good stability under both storage and physiological conditions. In contrast, US exposure induced a marked increase in 5‐FU release at both temperatures. Released drug concentrations were 3‐ to 4‐fold higher than the corresponding passive controls at all measured time points (^*^ *p* < 0.05, ^**^ *p* < 0.01, ^***^ *p* < 0.001, ^****^ *p* < 0.0001). Notably, drug release remained relatively constant throughout the experiment, with no substantial differences between the time points, indicating that US‐triggered release was maintained over the 24 h period. Overall, these findings demonstrate efficient US‐mediated drug release while preserving good stability in the absence of stimulation. Additionally, 5‑FU NDs stored for 3 months at 4°C (Figure 4k) exhibited baseline‐level passive leakage (0.288 ± 0.025 ppm after dilution 0.3:1), while US application induced substantial release (1.373 ± 0.043 ppm after dilution 0.3:1), representing a 4.5‐fold increase (^****^ *p* < 0.0001), confirming long‑term encapsulation stability.

### 2.8 In Vitro Evaluation of ND‐Mediated Mechanotherapy and Drug Uncaging

**FIGURE 5:**
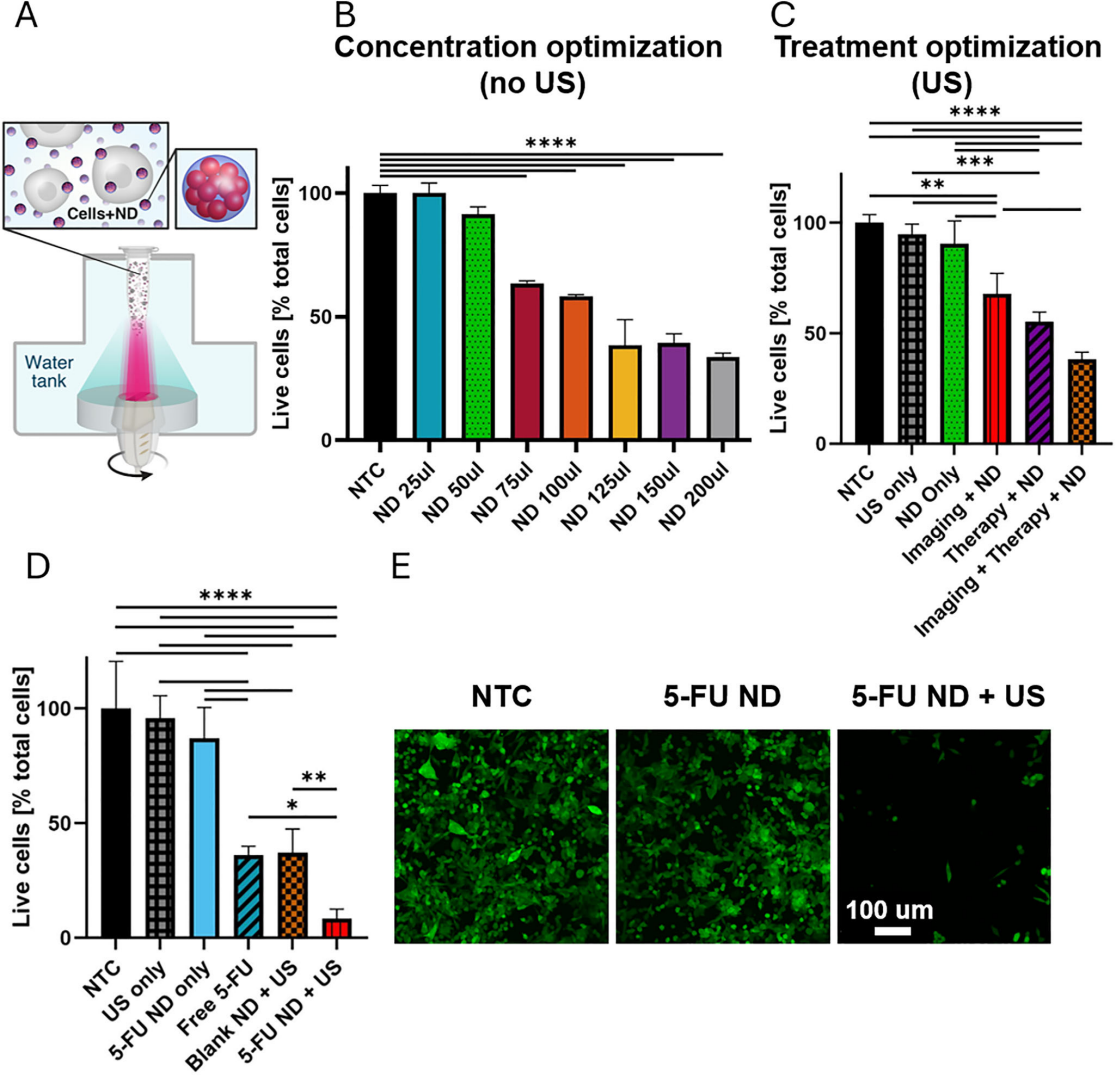
In vitro optimization and cytotoxicity of ND‐mediated mechanochemical therapy. (A) Schematic of the in vitro experimental setup. An Eppendorf containing a suspension of cancer cells mixed with NDs was positioned at the acoustic focal point of the US‐guided focused US system and exposed to US. Cell viability was assessed 72 h after treatment. (B) Effect of ND concentration on cell viability without US exposure. (C) Comparative analysis of cell viability of NTC, dual‐frequency US only, ND only, imaging transducer + ND, therapy transducer + ND, and the combined dual‐frequency + ND. (D) Quantitative viability analysis of live cells across therapeutic groups: NTC, US only, 5‐FU ND, Free 5‐FU, blank ND + US, and 5‐FU ND + US. The combined treatment demonstrates significant synergistic cytotoxicity. (E) Representative confocal microscopy images of GFP‐expressing 4T1 breast cancer cells following selected treatments. Scale bar: 100 µm. Data represent mean ± SD (C, D: *n* = 6, E: *n* = 3). Statistical significance was calculated using one‐way ANOVA with Tukey's multiple comparison test (^*^ *p* < 0.05, ^**^ *p* < 0.01, ^***^ *p* < 0.001, ^****^ *p* < 0.0001).

In vitro experiments were conducted in breast cancer cells (Figure 5a). First, we optimized the effect of blank NDs alone on cell viability. Cells were incubated with NDs for 72 h, and cell viability was measured. The ND concentration was 2.52 × 10^8^ ND/µL, and the ND volume varied between 25–200 µL. A dose‐dependent cytotoxicity was observed (Figure 5b). Low concentrations (25–50 µL) maintained >90% viability comparable to untreated controls (NTC; ^*^ *p* > 0.05), whereas an ND volume larger than 125 µL resulted in >60% cell death. These results are consistent with prior studies [43, 61]. Based on these results, 50 µL of blank NDs was selected as the optimal concentration for subsequent in vitro US experiments.

Before evaluating drug delivery effects, US‐triggered mechanotherapy was assessed using the optimized parameters and the dual‐frequency setup described in the uncaging experiments. Because the activation protocol involves two sequential US steps, we first evaluated the contribution of each step to cell viability. Cell viability was therefore assessed following ND vaporization induced by the imaging transducer alone, low‐frequency therapeutic US applied to NDs without prior vaporization, and the full dual‐frequency protocol combining both steps. Additional control groups included US‐only conditions without NDs and untreated cells. While all control groups maintained high viability (NTC: 100% ± 4.18%; US only: 94.68% ± 4.70%; blank NDs only: 90.46% ± 10.35%), ND+US combinations produced progressive reductions in viability. Imaging‐frequency US with NDs reduced viability to 67.99% ± 9.15% (^**^ *p* < 0.01 vs. NTC), therapeutic low‐frequency US with NDs to 55.35% ± 4.27% (^****^ *p* < 0.0001), and the full dual‐frequency protocol to 38.15% ± 3.32% (^****^ *p* < 0.0001), demonstrating a strong mechanotherapeutic effect (Figure 5c).

Prior to evaluating US‐triggered 5‐FU uncaging, the cytotoxic effect of free 5‐FU was first assessed. Free 5‐FU exhibited dose‐dependent cell cytotoxicity across concentrations of 9.6–76.9 µm with cell viability decreasing from 100% in the NTC to below 20% at 9.6 and 19.2 µm and under 10% at 38.4 and 76.9 µm (Figure S10), establishing the sensitivity of the cells to the chemotherapeutic agent within the in vivo therapeutic window. We next evaluated US‐triggered 5‐FU release from drug‐loaded NDs and compared the resulting combined chemo‐mechanical effect with the purely mechanical effect produced by blank NDs under the same US conditions (Figure 5d). The strongest cytotoxic effect was observed when US was applied to 5‐FU NDs, reducing cell viability to 8.42% ± 4.14% (^****^ *p* < 0.0001 vs. NTC; ^**^ *p* < 0.01 vs. blank NDs + US; ^*^ *p* < 0.05 vs. Free 5‐FU group). In comparison, activation of blank NDs by US reduced viability to 37.07% ± 10.35%, and free 5‐FU to 36.02% ± 3.86%, indicating that the additional cytotoxicity arises from US‐triggered drug release. Control conditions, including NTC (100.00% ± 20.51%), US alone (95.68% ± 9.78%), and only 5‐FU ND (86.93% ± 13.48%), did not have a significant impact on cell viability (Figure 5d). Confocal microscopy of GFP‐expressing breast cancer cells (Figure 5e) visually confirmed the progressive cytotoxic response across the treatment groups.

### 2.9 In Vivo Pharmacokinetics and Tumor Accumulation of NDs

Following the demonstration of combined mechanochemical efficacy in vitro, NDs were evaluated in a mice breast cancer model to characterize their in vivo pharmacokinetics, blood circulation, and tumor accumulation prior to therapeutic studies. Systemic administration in breast cancer‐bearing mice enabled quantification of ND blood clearance, biodistribution, and extravasation into solid tumors, providing a basis for selecting dosing and timing parameters for subsequent in vivo US therapeutic experiments. First, systemic pharmacokinetics and biodistribution of NDs were characterized. Although BDPg NDs exhibited peak fluorescence at 480/570 nm, in vivo biodistribution results showed significant autofluorescence from mouse tissues at these wavelengths. To overcome this, BDPfr NDs, with peak excitation/emission at 640/710 nm, were selected for in vivo imaging to minimize background signal.

**FIGURE 6:**
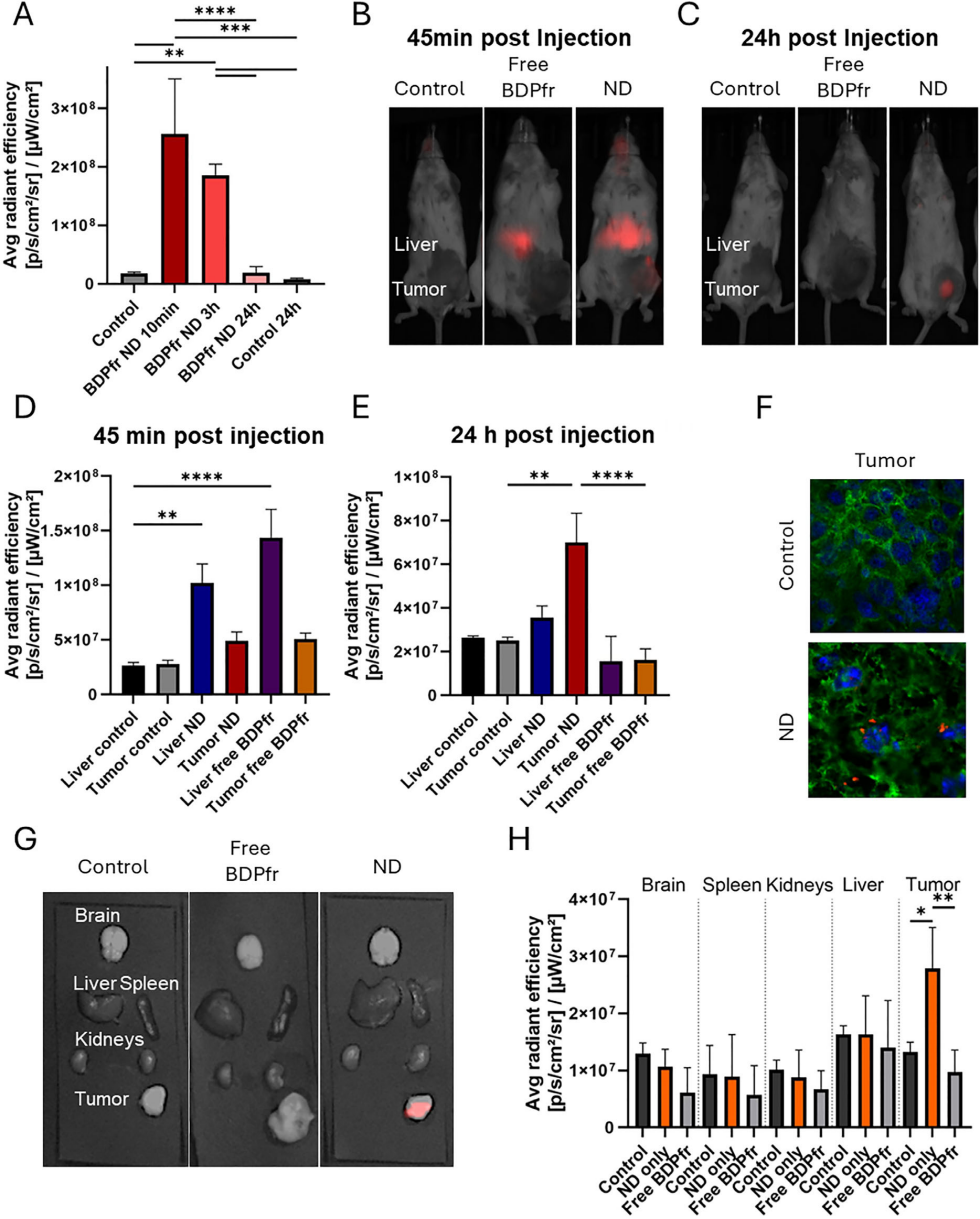
In vivo biodistribution and tumor accumulation of BDPfr ND. (A) Time‐dependent fluorescence intensity of BDPfr in blood samples 10 min, 3 h and 24 h post‐injection following administration of BDPfr ND, compared with NTC. (B, C) Representative whole‐body IVIS fluorescence imaging of tumor‐bearing mice at (B) 45 min, and (C) 24 h post‐injection. Groups include non‐injected control, free BDPfr dye, and BDPfr NDs. Regions of interest (ROIs) indicate liver and tumor locations. (D, E) Quantitative analysis of fluorescence intensity in liver and tumor regions at (D) 45 min, and (E) 24 h, highlighting statistically significant tumor retention in the ND‐treated group relative to controls and free dye. (F) Confocal microscopy of histological tumor sections from control and ND‐treated mice. Images display nuclei (DAPI, blue), cell membranes (WGA, green), and ND (BDPfr ND, red). The presence of intracellular red dots confirms deep tissue penetration and cellular uptake of the NDs. (G) Ex vivo fluorescence imaging of excised organs (brain, liver, spleen, kidneys, tumor) 24 h post‐injection, validating the biodistribution patterns observed in vivo. (H) Quantification of ex vivo radiant efficiency across harvested organs 24 h post‐injection. The analysis confirms preferential accumulation and high‐intensity signal retention within the tumor for the ND group compared to the control and free dye groups. Data represent mean ± SD, *n* = 3. Statistical significance was calculated using one‐way ANOVA with Tukey's multiple comparison test (^*^ *p* < 0.05, ^**^ *p* < 0.01, ^***^ *p* < 0.001, ^****^ *p* < 0.0001).

Blood pharmacokinetics of BDPfr NDs were quantified by IVIS fluorescence analysis of serial blood samples (10 min, 3 h, 24h post‐injection; Figure 6a). BDPfr NDs exhibited rapid signal increase (10 min: 2.56 × 10^8^ ± 9.41 × 10^7^ radiant efficiency vs. baseline 1.77 × 10^7^ ± 2.40 × 10^6^) followed by prolonged circulation (3h: 1.86 × 10^8^ ± 1.89 × 10^7^; 24h: 1.91 × 10^7^ ± 1.02 × 10^7^), indicating a blood circulation time exceeding 3 h.

Subsequently, IVIS imaging was used to track BDPfr ND and free BDPfr dye biodistribution in breast cancer‐bearing mice across 24 h, with representative time points shown at 45 min and 24 h. At 45 min post‐injection (Figure 6b,d), ND‐treated mice exhibited significantly elevated fluorescence in the liver (1.02  ×  10^8  ^±  1.73  ×  10^7^ average radiant efficiency) and tumor (4.91  ×  10^7^  ±  8.06  ×  10^6^). This represented a 3.8‐fold increase in liver fluorescence compared to controls (2.65  ×  10^7^  ±  2.97  ×  10^6^) and a 1.8‐fold increase in tumor fluorescence relative to controls (2.79  ×  10^7^  ±  3.61  ×  10^6^). ND‐mediated accumulation was comparable to that of free dye‐injected animals, which showed liver fluorescence of 1.44  ×  10^8^  ±  2.60  ×  10^7^ (5.4‐fold above control) and tumor fluorescence of 5.08  ×  10^7^  ±  5.51  ×  10^6^ (1.8‐fold above control). One‐way ANOVA with Tukey's multiple comparison test confirmed significant differences among group means at this time point (^**^ *p* < 0.01, ^****^ *p* < 0.0001). By 24 h post‐injection (Figure 6c,e), a clear divergence between formulations emerged. ND‐treated mice retained elevated tumor fluorescence (7.01  ×  10^7^  ±  1.34  ×  10^7^), 2.8‐fold above controls (2.51  ×  10^7^  ±  1.56  ×  10^6^) and 4.3‐fold greater than free dye (1.63  ×  10^7^  ±  5.03  ×  10^6^). In contrast, liver fluorescence in the ND group (3.56  ×  10^7^  ±  5.33  ×  10^6^) and free dye group (1.56  ×  10^7^  ±  1.14  ×  10^7^) were comparable to control (2.64  ×  10^7^  ±  8.49  ×  10^5^), indicating efficient hepatic clearance. One‐way ANOVA with Tukey's multiple comparison test again indicated significant differences among group means (**p < 0.01, ^****^ *p* < 0.0001).

Extended biodistribution kinetics over 90 h post‐injection (Figure S11) further elucidated the accumulation and retention behavior of BDPfr NDs. Within minutes of administration, ND‐treated mice displayed a rapid fluorescence increase in the tumor region, which remained significantly elevated and stable throughout the initial 4.5‐h imaging period (Figure S11a). Long‐term tracking revealed that while liver fluorescence peaked early and declined to near‐baseline levels by 24 h, tumor fluorescence continued to rise, reaching maximum intensity at approximately 55 h before declining slightly by 90 h while remaining above control levels (Figure S11b). The kinetic profile over 220 min (Figure S11c) further illustrates the distinct accumulation and retention dynamics for each experimental group, with NDs demonstrating sustained tumor localization compared to free dye. These results confirm strong and sustained tumor retention of BDPfr NDs compared to transient hepatic uptake.

To validate these in vivo observations, ex vivo analysis of harvested organs was performed at 24 h post‐injection (Figure 6g,h). This confirmed maximal tumor accumulation for NDs (2.79  ×  10^7^  ±  7.14  ×  10^6^), which was significantly higher than free dye (9.71  ×  10^6^  ±  3.88  ×  10^6^) and control (1.32  ×  10^7^  ±  1.71  ×  10^6^). These values represent a 2.9‐fold increase compared to free dye and a 2.1‐fold increase relative to control. In other organs, ND‐associated fluorescence was generally similar to background, except in spleen and kidneys, where values and variances were comparable to controls (Brown‐Forsythe tests, all *p* > 0.22), indicating no significant off‐target accumulation. One‐way ANOVA with Tukey's multiple comparison test for ex vivo data validated significant differences between the tumor ND group with tumor control (^*^ *p* < 0.05) or with tumor free dye group (^**^ *p* < 0.01).

To determine whether this macroscopic tumor retention of BDPfr NDs reflects deep tissue penetration and cellular uptake, we next examined tumor sections using confocal microscopy. Tumors collected 24 h post‐injection were processed for high‐resolution fluorescence imaging. Confocal microscopy (Figure 6f) showed penetration of BDPfr NDs within tumor sections, with red fluorescence (BDPfr ND) localized adjacent to cell nuclei (blue, DAPI) across multiple regions. Co‐staining with WGA Alexa Fluor 488 (green) highlighted cell membrane architecture and confirmed intracellular localization of NDs in merged images.

### 2.10 In Vivo Mechanotherapy in a Breast Cancer Mouse Model

**FIGURE 7:**
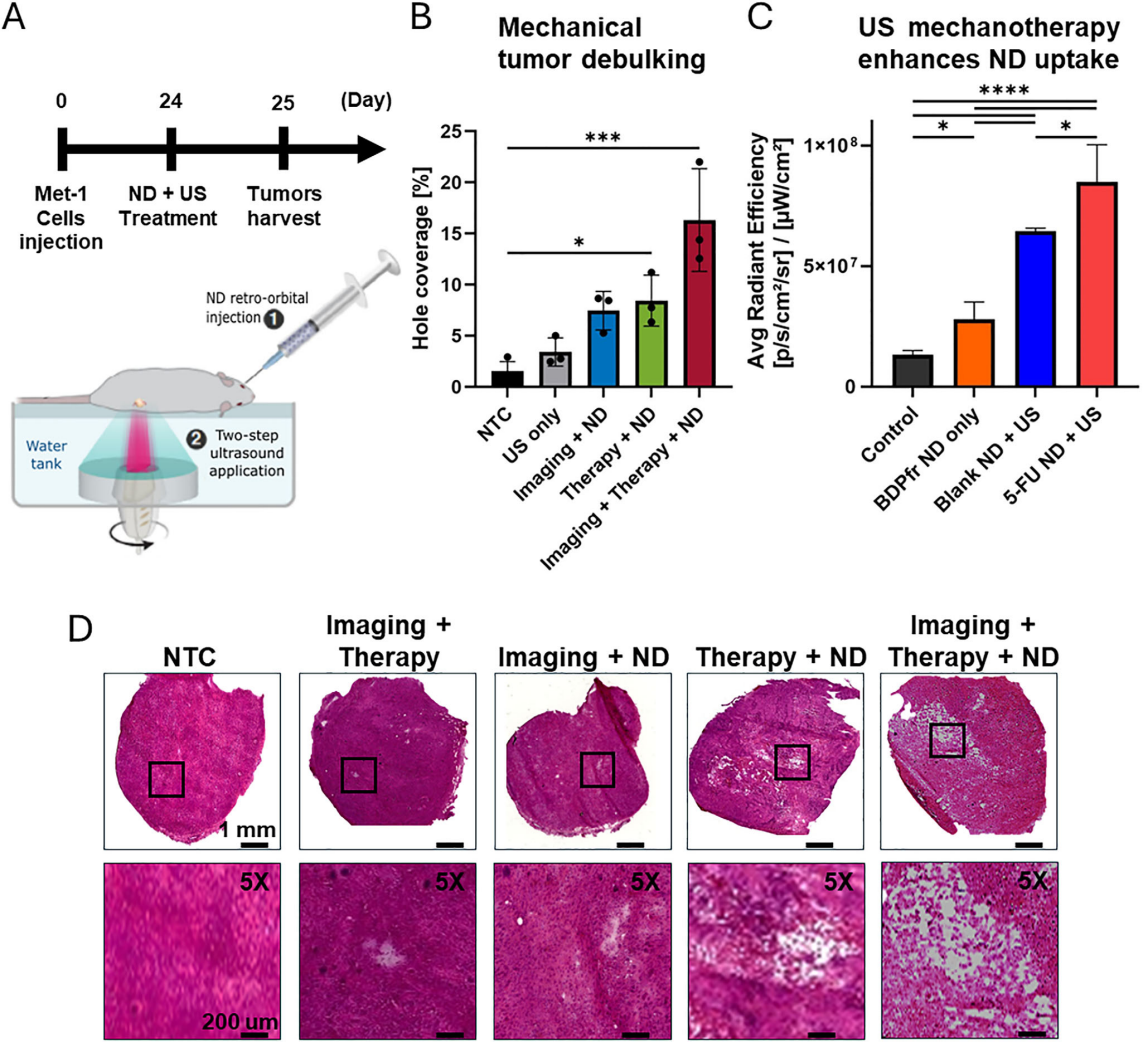
Two‐step US applied to blank ND induces mechanotherapy in vivo in a breast cancer tumor model. (A) In vivo experimental setup and treatment timeline. NDs were administered systemically, followed by dual‐frequency insonation of the tumor to induce mechanotherapy. (B) Quantification of histological damage, expressed as percentage of hole coverage (fractionated tissue area) across treatment groups: NTC, US only, imaging US + ND, therapy US + ND, and the full protocol (imaging + therapy + ND). The combined regimen induced maximal tissue fractionation. (C) Ex vivo IVIS analysis of average radiant efficiency in tumors following subsequent injection of BDPfr NDs. Groups include: naïve tumor control, tumor with BDPfr ND only, tumor pre‐treated with blank ND + US, and tumor pre‐treated with 5‐FU ND + US. Enhanced fluorescence was observed in tumors pre‐treated with mechanical ablation (blank ND + US), with significantly greater accumulation following mechanotherapy (5‐FU ND + US), indicating that therapeutic disruption progressively enhances vascular permeability and drug delivery. (D) Representative histological photomicrographs (H&E staining) of the treated tumors. Groups include NTC, US only, imaging + ND, therapy + ND, and the combined dual‐frequency + ND activation. Black squares indicate regions of successful tumor fractionation (mechanical ablation). Scale bars: 1 mm (whole tumor cross‐sections), 200 µm (5 × magnification). Data represent mean ± SD, *n* = 3. Statistical significance was calculated using one‐way ANOVA with Tukey's multiple comparison test (^*^ *p* < 0.05, ^**^ *p* < 0.01, ^***^ *p* < 0.001, ^****^ *p* < 0.0001).

To isolate mechanotherapy effects prior to combined treatment evaluation, blank NDs were tested with dual‐frequency US (Figure 7a). The 10 min post‐systemic blank ND administration (110 µL per mouse), mice received 2 min of dual‐frequency US. Histological analysis of tumor sections harvested 24 h post‐treatment revealed cavitation‐induced lesions in the ND + dual‐frequency group. In contrast, control groups, including untreated mice, US alone, or ND alone, exhibited minimal to no histological evidence of damage (mean lesion areas: 1.55%  ±  0.92% for untreated, 3.42%  ±  1.39% for US only). The dual‐frequency ND‐mediated group demonstrated significantly greater tissue fractionation, with lesion areas averaging 16.32%  ±  5.02% (Figure 7b,d, ^***^ *p* < 0.001). Following ND‐induced mechanotherapy, we hypothesized that the resulting vascular disruption would increase tumor vascular permeability and thereby enhance subsequent ND accumulation. To test this, BDPfr NDs were systemically injected 24 h after FUS treatment, and organ accumulation was quantified ex vivo using IVIS imaging (Figure 7c). Tumors in the BDPfr ND‐only group (2.79 × 10^7^ ± 7.14 × 10^6^ radiant efficiency) showed a 2‐fold increase relative to NTC (1.32 × 10^7^ ± 1.71 × 10^6^). Prior mechanotherapy (Blank ND + US) further increased tumor fluorescence to ∼5‐fold above control (6.44 × 10^7^ ± 1.27 × 10^7^). The highest signal was observed in tumors pre‐treated with combined mechanochemotherapy (5‐FU ND + US, 8.48 × 10^7^ ± 1.54 × 10^7^), corresponding to ∼7‐fold enrichment relative to NTC and ∼3‐fold relative to BDPfr ND alone. These results indicate that US‐mediated mechanical disruption, particularly when combined with chemotherapeutic delivery, increases tumor vascular permeability and promotes intratumoral ND accumulation.

### 2.11 Combined Mechanotherapy and Chemotherapy Drug Uncaging In Vivo

**FIGURE 8:**
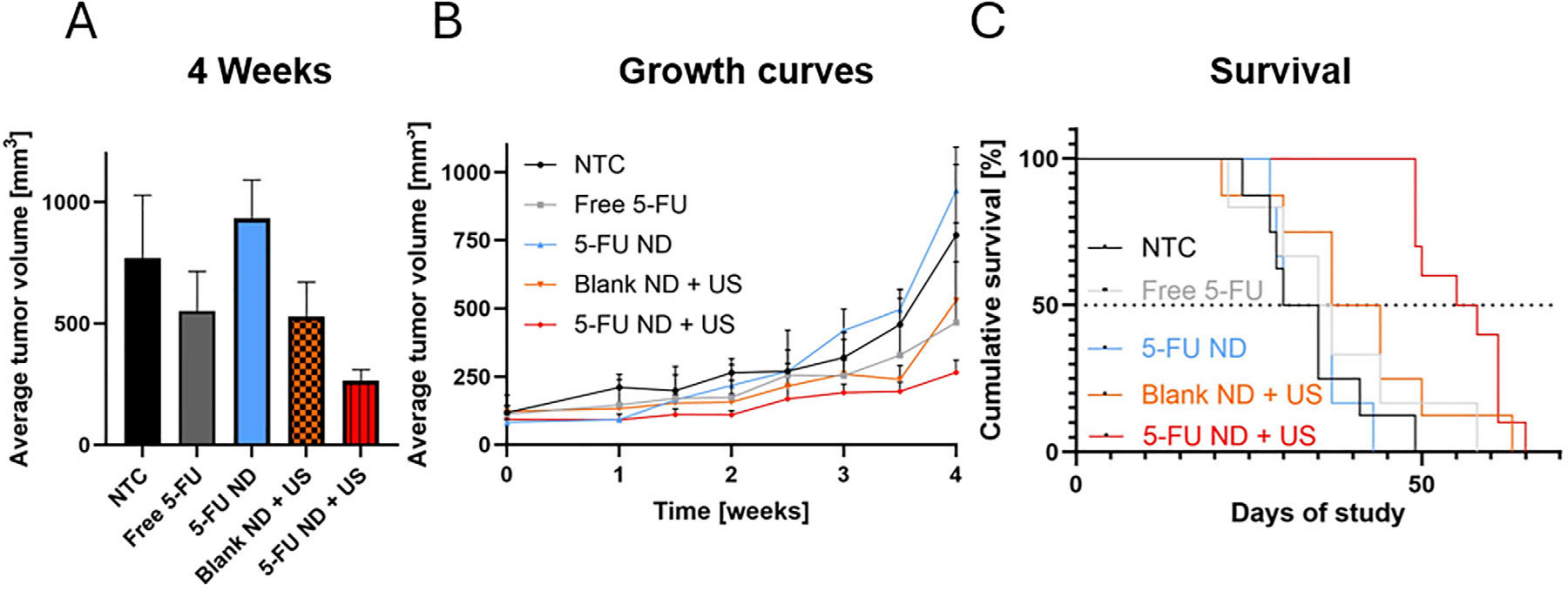
Therapeutic efficacy of ND‐mediated mechanotherapy and chemotherapy uncaging in vivo. (A) Average tumor volume measured in week 4 for the different treatments. (B) Tumor growth curves from day 1 (treatment initiation) to week 4 for NTC, Free 5‐FU, 5‐FU ND, blank ND + US, and 5‐FU ND + US groups. The 5‐FU ND + US group shows sustained tumor suppression over the treatment period. (C) Kaplan‐Meier survival curves displaying cumulative survival percentage over the experimental duration. The combined mechanochemical treatment (5‐FU ND + US) confers a significant survival advantage compared to all other groups. Data represent mean ± SD.

Lastly, the combined mechanotherapy and chemotherapy uncaging therapeutic effect was assessed by monitoring mice weight (Figure S12), tumor growth, and survival compared to four groups receiving weekly interventions: NTC, 5‐FU ND alone, free 5‐FU, blank ND + US, and 5‐FU ND + US. Tumor growth was monitored over four weeks to evaluate the therapeutic effect of combined mechanotherapy and chemotherapy uncaging (Figure 8a,b). Baseline tumor volumes (Figure 8b) were comparable across groups (NTC: 117.6 ± 71.75 mm^3^; 5‐FU ND: 81.93 ± 32.38 mm^3^; free 5‐FU: 112.58 ± 69.17 mm^3^; blank ND + US: 122.3 ± 51.30 mm^3^; 5‐FU ND + US: 92.98 ± 43.67 mm^3^). Over the treatment period, NTC and 5‐FU ND alone exhibited rapid tumor progression, reaching mean volumes of 768.2 ± 581.5 mm^3^ and 934.0 ± 417.8 mm^3^, respectively. In contrast, free 5‐FU and blank ND + US produced moderate tumor growth inhibition (448.5 ± 364.9 and 528.1 ± 376.6 mm^3,^ respectively), while the combined treatment (5‐FU ND + US) showed the strongest effect, limiting tumor growth to 265.6 ± 99.48 mm^3^ at 4 weeks (Figure 8a). This represented a 1.7‐fold reduction relative to free 5‐FU drug, a 2.0‐fold smaller tumor volume compared to blank ND + US, a 2.9‐fold reduction relative to NTC controls, and a 3.5‐fold decrease compared to 5‐FU ND alone.

These growth trends were reflected in survival outcomes (Figure 8c). Median survival was 32.5 days in the NTC group and 33.5 days in the 5‐FU ND group (without US), 36.0 days in the free 5‐FU group, while blank ND + US extended survival to 40.5 days. The longest survival was observed in the 5‐FU ND + US group, with a median survival of 56.5 days, corresponding to a 73.8% increase relative to NTC, a 68.7% improvement over 5‐FU ND alone, a 57% increase compared to free 5‐FU, and a 39.5% increase compared to blank ND + US. Survival analysis confirmed significant differences between groups (log‐rank test p = 0.0001), with additional support from the log‐rank trend test (*p* < 0.0001) and the Gehan‐Breslow‐Wilcoxon test (p = 0.0003). Monitoring of body weight throughout the survival study (Figure S12) revealed that no animal experienced weight loss exceeding 20% of its baseline weight. Together, these results demonstrate that US activation of 5‐FU NDs produces synergistic antitumor effects and significantly improves survival.

## 3 Discussion

This work aims to establish a robust solid tumor therapy platform by bringing together three elements into a single nanotherapeutic agent: microfluidic production of small and stable lipid NDs, core loading of therapeutics as a platform for drug delivery, and a dual‐frequency US protocol that couples ADV with low‐frequency cavitation and drug release. The microfluidic approach enabled precise control over ND size and concentration. Increasing the total flow rate produced smaller and more concentrated droplets, consistent with other microfluidic studies where faster mixing favors smaller droplet formation. Between the two PFC cores we tested, C_5_F_12_ and C_6_F_14_, C_5_F_12_ gave smaller droplets at a given flow rate but proved less stable over time and temperature, likely because its boiling point (29.5°C vs. 56°C of C_6_F_14_) is close to room and body temperature. C_6_F_14_ NDs, in contrast, maintained a stable size over 60 days at 4°C and remained liquid and intact at 37°C unless we applied focused US. Their more negative zeta potential (−29.8  ±  3.7 mV compared to −20.58 ± 2.95 mV) and lower size fluctuation further support the choice of C_6_F_14_ as the preferred core for in vivo work.

Our microfluidic production relies on a solvent‐shift self‐assembly mechanism. Lipids and PFC precursors are dissolved in the organic phase, and upon contact with the aqueous stream within the microfluidic channel, rapid ethanol diffusion drives spontaneous lipid shell formation around the insoluble PFC core. This approach, where lipids are dissolved in the organic phase rather than pre‐hydrated in the aqueous phase, follows established lipid nanoparticle formulation protocols [62, 63, 64, 65]. Following dialysis to remove residual ethanol, this method ensures efficient core encapsulation and stable droplet formation.

The acoustic experiments clarify how these physical properties translate into functional behavior. In tissue‐mimicking phantoms, C_6_F_14_ NDs remained inert at room temperature and at 37°C for MI values below 1.0, then gradually activated as the MI increased, with first signs of vaporization at MI around 1.58 and full ADV with strong contrast enhancement at MI ≥1.9 (Figure 2h,i). This threshold‐dependent activation is critical for clinical translation, as it ensures predictable vaporization only within targeted treatment zones while remaining safe during diagnostic imaging.

The core‐loading strategy for 5‐FU and BODIPY dyes is another important aspect. Rather than attaching the drug or dye to the shell, we dissolve both in the C_6_F_14_ phase before droplet formation. Many previously described PFC NDs carry drugs in the shell or in appended polymer layers, which can compromise stability and allow slow leakage over time. In our case, fluorination promotes preferential partitioning into the PFC core, where the payload remains retained until the liquid‐to‐gas phase transition is triggered by US.

The absence of a detectable fluorescence signal before US (Figure 3e) likely reflects the limited sensitivity and spatial resolution of optical microscopy for sub‐200 nm NDs. After ADV, the resulting MBs exceed the diffraction limit and are readily detected. ND visualization at these size scales is therefore better suited to electron microscopy.

Core encapsulation of the payload was confirmed by confocal microscopy of microdroplets (Figure S6), which showed distinct green fluorescence (BDPg) confined to the PFC core interior, bounded by an outer red fluorescence signal (DiI) in the lipid shell, thus providing direct visual evidence for the core‐localized encapsulation. DLS measurements further confirmed that both payloads remained molecularly dispersed within the PFC phase without detectable aggregation (Figure S7), and encapsulation efficiencies exceeded 84% for both BDPg and 5‐FU, demonstrating effective cargo loading and preservation of ND structural integrity.

Our PFC‐core loading strategy is directly applicable to molecules containing fluorine atoms. Notably, approximately 15%–20% of marketed small‐molecule drugs contain fluorine, and in 2025, 14 of the 29 FDA‐approved small‐molecule drugs were fluorinated [53]. For non‐fluorinated payloads, fluorine substitution is a well‐established strategy in medicinal chemistry that typically improves lipophilicity, metabolic stability, binding affinity, and bioavailability [54, 66]. Fluorination has been successfully applied across diverse therapeutic classes, including small molecules, nucleic acids, peptides, proteins, and carbohydrates, without compromising bioactivity. The large and growing library of clinically approved fluorinated therapeutics provides a broad range of candidate payloads for this platform, enabling selection of compounds best suited for a given therapeutic application.

Importantly, the fluorinated payloads remained stably retained within the core under both storage (4°C) and physiological (37°C) conditions, with minimal passive leakage observed over extended periods, including after three months of storage (Figure 4k).

Notably, the fluorescence signal originates from BODIPY within the released PFC phase, not from free BODIPY dissolved in buffer.

Following dual‐frequency US activation, substantial payload release was achieved, producing approximately 2.8‐fold (Figure 4c) and 4‐fold (Figure 4h) increases in released BDPg and 5‐FU, respectively. These findings support the proposed mechanism in which fluorination promotes preferential partitioning of the payload into the PFC core, allowing retention until ADV and subsequent cavitation trigger release. Beyond drug delivery, multispectral IVIS characterization demonstrated that the BODIPY‐loaded NDs possess distinct and well‐resolved fluorescence signatures, enabling optical tracking and multiplexed imaging of different ND populations in heterogeneous biological environments (Figure 3d). Together, these results highlight the potential of core‐loaded PFC NDs as stable, externally controlled theranostic agents that overcome limitations associated with conventional shell‐based loading approaches.

The biological data show how these physical and acoustic properties translate into therapy. In vitro studies with 4T1 breast cancer cells were essential for evaluating blank ND cytotoxicity prior to US‐mediated detonation. ND concentrations ranging from 25–200 µL were tested, and concentrations above 50 µL (1.26 × 10^10^ NDs / 50 µL) reduced cell viability (Figure 5b), a concentration‐dependent toxicity likely attributable to the phospholipid shell components. At high phospholipid concentrations, in vitro cytotoxicity has been reported due to membrane disruption or metabolic interference [67].

It is important to note, however, that cells in culture are more sensitive to such effects than in the complex biological environment, where biological fluids and clearance mechanisms may mitigate direct nanoparticle‐cell interactions. At the chosen concentration, blank ND and US alone had no significant effect on cell viability. Combining ND with dual‐frequency US, however, reduced cell viability to about 38%, a 62% reduction. These observations suggest that the dual‐transducer protocol, inducing ADV via the imaging transducer (maintaining MI below 1.9 at acoustic pressures over 2 MPa) followed by low‐frequency inertial cavitation, produces meaningful mechanical disruption even without drug present, consistent with reports of mechanical tissue fractionation in histotripsy and related techniques. The low‑frequency transducer was selected over a high‑frequency one to promote inertial cavitation of the gas bubbles generated during ADV, as lower frequencies (e.g., hundreds of kHz) lower the inertial cavitation threshold compared to MHz frequencies, enabling more efficient bubble collapse, microjet formation, and mechanical disruption at clinically feasible acoustic pressures. Importantly, although the two transducers are physically operated simultaneously during treatment, the therapeutic effect proceeds via a two‐step mechanism: vaporization of NDs into MBs followed by inertial cavitation and collapse, which triggers payload release. The synergistic cytotoxic effect is achieved only when both transducers are applied together.

When 5‐FU was encapsulated in the NDs and US was applied in the presence of tumor cells, viability fell further to 8.41%, much lower than what we saw with passive 5‐FU ND exposure (Figure 5d). This pattern supports a synergistic effect, a combination of mechanical damage from the cavitation and controlled chemotherapy effect.

In vivo, a consistent picture emerged. BDPfr NDs showed higher and more persistent tumor signal than free BDPfr dye, both in IVIS in vivo and ex vivo imaging (Figure 6). At 24 h, tumor fluorescence in ND‐treated animals was 2.87‐fold higher than in those given free dye, whereas free dye signals had largely returned to baseline in most tissues (Figure 6c,e). This indicates that the ND formulation improves both delivery to, and retention in, the tumor, as expected for a nanoscale carrier operating under the EPR effect. It is well‐established that endothelial permeability increases in pathological states such as tumors and infarcted areas, enabling extravasation of large molecules and particles (10–500 nm, e.g., micelles, liposomes) into the interstitial space via EPR [13, 14, 15, 68]. Nanoparticles with hydrodynamic diameters of 100–400 nm are considered optimal for passive tumor targeting through EPR, balancing vascular extravasation with retention while exceeding the renal clearance threshold (>40 kDa or ∼10 nm diameter), as glomerular sieving restricts first‐pass kidney elimination of smaller species. In this context, the strong BDPfr ND signal persisting in tumors 24 h post injection reflects robust tumor targeting, ND stability in circulation, and sustained intratumoral retention, while the relatively low off‐target signal in most organs supports a favorable biodistribution profile. Confocal microscopy (Figure 6f) goes a step further by showing that these NDs not only accumulate in the tumor but also reach deep tissue regions and intracellular compartments, indicating that the macroscopic retention measured by IVIS corresponds to genuine intra‐tumoral distribution and cellular uptake rather than surface‐restricted binding.

We hypothesize that several formulation features may contribute to the prolonged circulation time and biodistribution observed in this study. The PEGylated lipid shell (DSPE‐PEG2k) is known to prolong circulation and reduce reticuloendothelial system uptake, as demonstrated with clinically approved liposomes such as Doxil, which exhibit extended half‐lives compared to non‐PEGylated formulations [69, 70, 71]. Additionally, recent studies have shown that nanoparticle composition can influence biodistribution, with certain lipid formulations reducing accumulation in clearing organs such as the liver [72]. The liquid PFC core and small particle size (<200 nm) may further modulate hepatic interactions.

To isolate mechanotherapy effects prior to combined treatment evaluation, blank NDs were tested with dual‐frequency US and H&E staining of tumor sections (Figure 7) revealed cavitation‐induced lesions in the ND + dual‐frequency group compared to other groups (mean lesion areas: 1.55% ±  0.92% for untreated, 3.42%  ±  1.39% for US only, 16.32%  ±  5.02% for ND + dual‐frequency group). The extent of mechanotherapy is determined by the combined effects of the US focal spot and the intratumoral distribution of the NDs, which depends on local vascular perfusion and extravasation.

The survival and tumor growth data confirm that these physical and imaging advantages have real therapeutic consequences (Figure 8). Weekly treatment with 5‐FU NDs plus dual‐ frequency extended median survival from about 32.5 days in controls to 56.5 days (73.8% increase). Free 5‐FU drug or Blank NDs with US gave an intermediate benefit, consistent with a contribution from purely chemotherapy or mechanical damage, but the best outcome came from the combined chemomechanical therapy, demonstrating that our platform combines effective tumor targeting, deep tissue penetration, and potent mechanochemical therapy within a single US‐controllable carrier. The platform's clinical translation potential is supported by several factors: microfluidic synthesis is compatible with GMP‐scale production, PFCs have decades of clinical safety data, all acoustic parameters comply with FDA guidelines, and the dual‐ frequency system was assembled using off‐the‐shelf transducers. The ability to achieve therapeutic efficacy with reduced systemic drug exposure addresses a major limitation of conventional chemotherapy, and the demonstrated capacity for multispectral optical tracking and robust tumor‐selective retention and intracellular delivery further supports the use of these core‐loaded NDs as a modular theranostic platform. Although passive cavitation detection was not performed in the present study, future investigations employing broadband acoustic detection could directly confirm inertial cavitation and bubble collapse during therapeutic US application [73].

In terms of future directions, while this study demonstrated efficacy in a breast cancer model, the platform's versatility could extend to other indications and additional cancer types. NDs have been shown to enable blood‐brain barrier opening via acoustic activation [74, 75]. It would therefore be valuable to explore this platform for targeted drug delivery in brain cancer and other neurodegenerative diseases such as Alzheimer's and Parkinson's disease. In addition, the mechanochemical mechanism demonstrated here could be adapted for targeted therapy in inflammatory diseases, potentially reducing systemic immunosuppression associated with current treatments. Moreover, the tumor fractionation achieved by this approach may enhance immune infiltration and activation, suggesting that combining this method with immunotherapy represents a promising future direction.

## 4 Conclusion

In summary, this study establishes an acoustically programmable ND platform that integrates nanoscale drug delivery, efficient core loading, and mechanochemical therapy. Microfluidic nanoassembly enables the reproducible production of stable sub‐200 nm NDs, while the physicochemical compatibility of fluorinated payloads with the liquid PFC phase allows efficient core loading with minimal passive release. Following systemic administration and tumor accumulation, dual‐frequency US couples ADV with low‐frequency cavitation, transforming the NDs into locally activated therapeutic agents that combine mechanical tissue fractionation with on‐demand drug release. This integrated approach enhanced cytotoxicity in vitro and produced substantial tumor fractionation, increased intratumoral delivery, and superior tumor control in vivo. Most notably, 5‐FU NDs combined with US extended median survival to 56.5 days, a 73.8% increase compared with untreated controls. Together, these findings establish a mechanochemical nanomedicine strategy that combines drug transport, controlled release, and mechanical tumor disruption within a single acoustically activated nanoscale platform.

## 5 Materials and Methods

### 5.1 Chemicals and Cell Culture

The preparation of 1,2‐Distearoyl‐sn‐glycero‐3‐phosphocholine (DSPC, 850365P), and 1,2‐distearoyl‐*sn*‐glycero‐3‐phosphoethanolamine‐*N*‐[meth‐oxy(polyethyleneglycol)‐2000] (ammonium salt) (DSPE‐PEG2K, 880120P) were purchased from Avanti Polar lipids (Alabaster, AL, USA). Ethanol absolute (cat. no. 000525052100) was provided by Bio‐Lab. PBS(−/−) 1 × (phosphate‐buffered saline without calcium and magnesium) was purchased from Sartorius (Cat# 02‐023‐1A), PBS(+/+) 1 × (phosphate‐buffered saline with calcium and magnesium) was purchased from Sartorius (Cat# 02‐020‐1A), Glycerol anhydrous was purchased from Bio‐Lab, Propylene Glycol from Sigma–Aldrich (1,2‐propanediol, Cat# 398039–500, St. Louis, MO, USA), BODIPY 493/503 (BDPg, 99%, CAS Number: 121207‐31‐6), Wheat germ agglutinin (WGA) Alexa Fluor 488 (W11261, Invitrogen) and 5‐Fluorouracil (F6627, CAS Number: 51‐21‐8) were purchased from Sigma–Aldrich (St. Louis, MO, USA), BDP TR‐X‐NHS ester (BDPr, 95%, CAS Number: 197306‐80‐2) and BDP 630/650‐X‐NHS ester (BDPfr, 95%, CAS Number: 2213445‐35‐1) were purchased from Lumiprobe, Agarose powder was purchased from Thermo Fisher (A10752, Thermo Fisher Scientific, OR, USA), Dulbecco modified Eagle medium (DMEM, Biological Industries [BI] Ltd., Cat # 01‐055‐1A, Kibbutz Beit‐Haemek, Israel), RPMI 1640 medium w/ L‐Glutamine was provided by Biological Industries Israel Beit‐Haemek Ltd. (Cat # 01‐100‐1A), TrypLE Express dissociation reagent (Gibco Corp, 12604‐013, Grand Island, NY, USA), 4T1 cells were acquired from ATCC (CRL‐2539) and Met‐1 mouse breast carcinoma cells were a gift from Prof. Jeffrey Pollard, University of Edinburgh, Edinburgh, UK, and Prof. Neta Erez, Tel Aviv University, Tel Aviv, Israel. Cell cultures were maintained in an incubator at 37°C in a controlled atmosphere (5% CO_2_, 95% humidity) using the culture media required by the producer using T25 and T75 flasks. All chemicals were used without further purification.

### 5.2 Microfluidic Fabrication of NDs

The preparation of C_5_F_12_ and C_6_F_14_‐core lipid‐shell NDs was achieved using a two‐step microfluidic fabrication process utilizing the NanoAssemblr platform (v 1.5, Precision NanoSystems Inc., Vancouver BC, Canada) or by using a Herringbone chip (LTF‐012.00‐4264, Darwin Microfluidics, France). In microfluidic technology, aqueous and organic phases are positioned in distinct streams within microchannels and are mixed through controlled laminar flow, where diffusion across their interface enables precise and efficient blending without turbulence. The aqueous phase was prepared by combining PBS(−/−) (pH 7.4), glycerol, and propylene glycol in an 8:1:1 v/v ratio. The mixture was then subjected to vortex mixing for 2 min. Following this, the mixture was sonicated in a water bath at 62°C for 15 min until it became homogeneous. The organic phase consisted of DSPC and DSPE‐PEG2K (9:1 molar ratio) dissolved in ethanol (1.2 mg/mL lipid concentration), sonicated at 62°C until clear. The aqueous and organic solutions were maintained at 5°C ± 3°C before synthesis. PFC (C_5_F_12_ or C_6_F_14_) was added to the cold organic solution at 10 µL/mL ethanol just before use in the microfluidic system. Then, the two cold mixtures were vigorously stirred for 2 min using a vortex mixer until a homogeneous solution was achieved. Each mixture was then transferred into individual syringes and connected to the inlets of a microfluidic chip for further processing.

To prepare encapsulated NDs, the process follows the same protocol as for regular NDs, with an additional step to incorporate the payload. For dye‐loaded NDs (BDPg, BDPr, and BDPfr), the fluorescent dye is dissolved in the PFC (either C_5_F_12_ or C_6_F_14_) at a concentration of 0.5 mg/mL prior to its addition to the ethanol solution; alternatively, the fluorescent dye can be dissolved in the pre‐cooled ethanol solution containing the lipids and PFC at 0.05 mg/mL. Similarly, for drug‐loaded NDs, the therapeutic chemotherapy (5‐Fluorouracil) is dissolved in the pre‐cooled ethanol solution containing the lipids and PFC at 0.1 mg/mL.

Two types of microfluidic‐generated NDs were compared. Nanoassembly ND synthesis was performed using the NanoAssemblr chip (Precision Nanosystems, 1146‐039) with a TFR of 12 mL/min and a FRR of 3:1 (aqueous: organic), using the microfluidic mixing device NanoAssemblr. For the formation of other NDs under lower TFRs (100‐2750 uL/min, FRR 3:1), a microfluidic system that included a Herringbone Mixer Glass Chip and two NE‐300 InfusionONE Syringe Pumps (NE‐300‐ES, Darwin Microfluidics, France) was used.

Following microfluidic synthesis, the suspensions were purified by dialysis using a Pur‐A‐Lyzer Maxi Dialysis Kit (12‐14 kDa MWCO, 3 mL capacity) under gentle stirring at 4°C. For standard preparations, dialysis was performed against phosphate‐buffered saline (PBS−/−, pH 7.4) for 24 h with buffer replacements at 3 and 6 h (1:200 v/v sample‐to‐PBS ratio) to remove residual ethanol, unencapsulated phospholipids, fluorescent molecules, and free therapeutic drugs. For TEM and E‐SEM samples, deionized water was substituted as the dialysis medium (22 h total, with buffer changes at 3 and 6 h). Post‐dialysis, all samples were stored at 4°C.

#### 5.2.1 Formulation Example

The aqueous phase comprised PBS (32 mL), glycerol (4 mL), and propylene glycol (4 mL), totaling 40 mL. The organic phase consisted of DSPC (22.7 mg), DSPE‐PEG2k (2.5 mg), EtOH (10 mL), and C_5_F_12_ (100 µL). Microfluidic parameters included, for example, organic flow rates of 25, 75, and 125 µL/min, with corresponding aqueous flow rates of 75, 225, and 375 µL/min, resulting in FRR of 3:1 and TFR of 100, 300, and 500 µL/min.

### 5.3 ND Characterization

The size distribution and concentration of the NDs were characterized using Nanoparticle Tracking Analysis (NTA, NanoSight NS300, Malvern Panalytical, UK) with software version NTA 3.4 Build 3.4.4, Dynamic Light Scattering (DLS, Zetasizer Ultra, Malvern Panalytical, UK), and, for selected samples, the Prometheus Panta system (NanoTemper Technologies, Germany). Zeta potential was measured using Zetasizer Ultra (DLS, Zetasizer Ultra, Malvern Panalytical, UK). The morphology of the NDs was further examined using Transmission Electron Microscopy (TEM, JEM 1400plus), and Environmental Scanning Electron Microscopy (E‐SEM) was performed using the Quanta FEG 650 (Thermo Fisher Scientific, USA) at an accelerating voltage of 30 kV, equipped with a Solid‐State Detector (SSD) (Thermo Fisher Scientific, USA).

The physical stability of the NanoAssemblr‐NDs in suspension was evaluated by monitoring their size distribution over time. To assess the stability of the NDs under storage conditions, the droplets were stored at 4°C for up to one month (with blank C_5_F_12_ and C_6_F_14_ ND samples extended to two months for additional evaluation). The ND suspensions were 10‐fold diluted with deionized water and analyzed using a Zetasizer Ultra DLS instrument. To evaluate the stability of C_5_F_12_ and C_6_F_14_ NDs under physiological conditions, the suspensions were maintained at 25°C, 37°C, and 42°C in a Panta DLS device for up to one hour. Size distribution measurements were taken at regular intervals to monitor changes in droplet stability under these conditions.

### 5.4 Ultrasound Imaging of ND Activation Thresholds and MB Inertial Cavitation

ND activation and MB inertial cavitation were monitored using an L7‐4 linear array transducer (Philips, ATL, 5.5 MHz center frequency, 128 elements) mounted on a programmable Vantage 256 ultrasound system (Verasonics Inc., Redmond, WA, USA). For ND activation, the same transducer delivered focused acoustic pressures and acquired B‐mode images pre/post‐activation. For inertial cavitation, a spherically focused, single‐element therapeutic transducer (H149, Sonic Concepts, Bothell, WA, USA, 105 kHz center frequency, 65 mm focal length) was applied, while the L7‐4 transducer acquired B‐mode images post‐cavitation. The therapeutic transducer was driven by a TPO‐200 amplifier (Sonic Concepts) and connected through a matching network (Sonic Concepts).

Tissue‐mimicking agarose phantoms (1.5% agarose in deionized water) contained cylindrical inclusions (3D‐printed mold: 65 × 25 × 20 mm outer dimensions, 15 mm tall × 8 mm diameter central rod) for ND activation and in vitro experiments, filled with ND suspension (10 µL NDs in 1 mL degassed PBS (−/−)). Phantoms were positioned with the ND inclusion at the transducer focal point. Experiments characterized acoustic vaporization thresholds and thermal stability at 25°C, 37°C, and 42°C for up to 2 h using MI 1.5‐2.1. For MB cavitation, the experiments were performed using MI 1.9 for ND activation (5.5 MHz) and MI 0.84 for inertial cavitation (105 kHz, 0.3 ms burst length, 30 ms total length, 60 s duration).

For each temperature, samples were stored in separate 0.5 mL tubes, and three independent samples were measured per time point (*n* = 3 per temperature). ND‐to‐MB phase transition and inertial cavitation were detected as echogenicity enhancement due to gas‐core acoustic impedance mismatch, visualized in real‐time B‐mode imaging. All experiments were performed in triplicate.

### 5.5 Dual‐ Frequency Focused Ultrasound System

The US setup was adapted from our previous study [43, 76] and consisted of a dual‐frequency system integrated within a water tank. A spherically focused, single‐element therapeutic transducer (H149, 105 kHz center frequency, 65 mm focal length) was coaxially aligned with a 1D rotating imaging probe (IP104, Sonic Concepts, Bothell, WA, USA) positioned at its base. The therapeutic transducer was driven by a TPO‐200 amplifier and connected through a matching network. The 128‐element imaging probe (13.5 mm elevation × 28.2 mm aperture) was controlled by the Verasonics system and mounted on a motorized rotary stage (RTY‐IP100, Sonic Concepts) capable of ± 180° rotation, with motion parameters governed by custom MATLAB scripts.

Transducer calibration was performed using a 0.2 mm needle hydrophone (NH0200, Precision Acoustics, Dorchester, U.K.) and a 3D positioning system (Newport motion controller ESP 300). The full‐width at half‐maximum (FWHM) of the focal zones was characterized as 1.2 mm (x) × 2.6 mm (y) × 23 mm (lateral) for the imaging probe and 10 mm (lateral) × 40 mm (axial) for the therapeutic transducer. The PNP for both transducers was calibrated in situ. For ND vaporization, the imaging probe delivered 2‐cycle sinusoidal pulses (3.5 MHz, MI 1.67) with single‐focus steering and rotation to cover the treatment volume. This probe also facilitated in vivo tumor imaging and positioning within the focal area.

### 5.6 Fluorescent Labeling, Encapsulation Efficiency and Release Characterization

Fluorescent NDs encapsulated fluorine‐functionalized BODIPY derivatives in green (BDPg), red (BDPr), or far red (BDPfr). To assess release kinetics, BDPg NDs were evaluated using a SPARK multimode microplate reader (Tecan Group Ltd., Männedorf, Switzerland) in fluorescence mode. Experiments were performed in 96‐well plates with data acquisition and analysis via SPARKControl software. For the encapsulation efficiency experiment, the supernatant solutions were collected and compared to whole solutions treated with acetonitrile to disrupt the lipid shell and release the encapsulated drug. The collected samples were diluted 10‐fold with DI and filtered through a 0.22‐µm membrane filter. Fluorescence intensity was then measured to quantify the extent of fluorophore release using the microplate reader and spectrophotometer, with a total measurement volume of 200 µL per well in a 96‐well format. Encapsulation efficiency was calculated using the following Equation (1):

(1) $$\mathrm{Encapsulation}\;\mathrm{efficiency}\;(\mathrm{EE}\%)=(\frac{T0_{\mathrm{whole}}-T0_{\sup}}{T0_{\mathrm{whole}}}x100\%)$$

where T0_whole_ is the total amount of payload measured after complete ND disruption with acetonitrile, and T0_*sup*_ is the amount of unencapsulated payload measured in the pre‐treatment supernatant. For US‐triggered release experiments, US‐triggered release was evaluated using a dual‐ frequency system incorporating a 3.5 MHz imaging transducer (MI 1.67) and a 105 kHz therapy transducer (MI 0.84) operating concurrently. Top supernatant solutions were collected before and after US exposure, diluted 10‐fold with DI, and filtered through a 0.22‐µm membrane filter; then the fluorescence intensity was measured to quantify the extent of fluorophore release by microplate reader and spectrophotometer, using a total measurement volume of 200 µL per well in a 96‐well format. The change in fluorescence intensity between pre‐ and post‐US treatments represented the release of fluorescent payload. Calibration was performed using free dye standards at concentrations ranging from 0.0 to 0.6 µm in Chloroform (0.0, 0.1, 0.143, 0.2, 0.3, 0.4, 0.5, and 0.6 µm). The excitation and emission wavelengths were 493 ± 5 nm and 513 ± 5 nm, respectively.

To determine encapsulation stability (passive and active release via US), samples of BDPg ND solution were stored at 4°C or 37°C for 1, 2, 4, 6, or 24 h. Supernatants were collected before and after US application, diluted 10‐fold with DI, and filtered through a 0.22‐µm membrane filter. Fluorescence intensity was then measured using a microplate reader and spectrophotometer to quantify the extent of fluorophore release, with a total measurement volume of 200 µL per well in a 96‐well format. The change in fluorescence intensity between pre‐ and post‐US treatments, as well as the release profile over time, represented the release of the fluorescent payload. Experiments were performed in triplicate.

### 5.7 5‐Fluorouracil (5‐FU) Chemotherapy Encapsulation and Release Quantification

To validate the differential solubility of 5‐FU in C_6_F_14_ vs. aqueous media, 5‐FU was dissolved in either C_6_F_14_ or DI water at a concentration of 0.4 mg/mL. Following vigorous mixing, solutions were filtered through a 0.22‐µm membrane filter to remove undissolved particulates. 5‐FU concentration can be estimated by UV‐spectrophotometer [77]. Fluorescence intensity was evaluated using a Spark multimode microplate reader (Tecan Group Ltd., Männedorf, Switzerland). Samples (pure DI water, pure C_6_F_14_, 5‐FU in DI, and 5‐FU in C_6_F_14_) were loaded into UV‐transparent 96‐well plates while scanned wavelengths were within UV spectrum, and data were acquired using SparkControl software. A preliminary full‐spectrum scan was performed to identify optimal peak wavelengths (excitation: 240–295 nm, emission: 310 ± 5 nm), followed by quantitative endpoint analysis using optimized parameters (excitation: 265 ± 20 nm, emission: 310 ± 20 nm). All measurements were performed in triplicate, and background signals from pure solvents were subtracted during analysis.

To assess release kinetics and encapsulation efficiency, 5‐FU NDs were evaluated using high‐performance liquid chromatography (HPLC, Agilent 1260 Infinity Series) equipped with a photodiode array UV–vis detector. For the encapsulation efficiency experiment of 5‐FU NDs (1.18  ×  10^11^ NDs/mL), the supernatant solutions were collected and compared to whole solutions treated with acetonitrile to disrupt the lipid shell and release the encapsulated drug. The collected samples were diluted 0.3:1 with DI and filtered through a 0.22‐µm membrane filter, and transferred to 2 mL HPLC vials prior to injection.

Quantification of 5‐FU release from 5‐FU NDs (1.18  ×  10^11^ NDs/mL) at 25°C pre and post US activation was conducted using HPLC. For passive release, supernatants were collected and diluted 100‐fold with deionized water, prefiltered through 0.22 µm membranes, and transferred to 2 mL HPLC vials prior to injection. For active release, supernatants collected after US exposure (under the same US parameters as the fluorescence release experiment with dual‐frequency set‐up) were diluted 100‐fold with deionized water, prefiltered through 0.22 µm membranes, and transferred to 2 mL HPLC vials prior to injection. Quantification of 5‐FU release from 5‐FU NDs (1.18  ×  10^11^ NDs/mL) during 24 h at 4°C or 37°C (passive release), or active release by US (also after solution storage at 4°C or 37°C up to 24 h) was conducted using HPLC. For passive release, supernatants collected after storage at 4°C or 37°C for 1, 2, 4, 6, or 24h, and were diluted 0.3:1 with deionized water, prefiltered through 0.22 µm membranes, and transferred to 2 mL HPLC vials prior to injection. For active release, after storage at 4°C or 37°C for 1, 2, 4, 6, or 24 h, supernatants collected after US exposure (under the same US parameters as the fluorescence release experiment with a dual‐frequency set‐up) were diluted 0.3:1 with deionized water, prefiltered through 0.22 µm membranes, and transferred to 2 mL HPLC vials prior to injection.

For all experiments, a 50 µL sample was injected into a Fast‐Poroshell 120 EC‐C18 column (100 mm × 4.6 mm, 4 µm, Agilent Technologies, USA) maintained at 30°C. The mobile phase was an isocratic mixture of 4% HPLC‐grade methanol (Bio‐Lab Ltd., Israel) and 96% deionized water containing 0.1% acetic acid (Merck KGaA, Germany), at a flow rate of 0.8 mL min^−1^. Detection was performed at 254 nm with a retention time of 2.2 min, consistent with established analytical protocols [78, 79]. Calibration curves were generated using free 5‐FU standards at concentrations ranging from 0.005 to 0.1 ppm for release study at 25°C under dilution of 100‐fold, and from 0 to 4 ppm (0, 0.1, 0.2, 0.5, 1, 2, and 4 ppm) for encapsulation efficiency, release at 4°C or 37°C, and stability experiments under dilution of 0.3:1. All measurements were performed in triplicate.

### 5.8 Transmission Electron Microscopy

Transmission Electron Microscopy (TEM) was conducted using the JEM 1400plus transmission electron microscope (Jeol, Japan). Samples were adsorbed onto Formvar/carbon‐coated grids and stained with 2% aqueous uranyl acetate prior to imaging. Images were captured using the SIS Megaview III camera and iTEM imaging platform (Olympus, Japan). For sample preparation (negative staining approach), 10 µL samples were diluted with DI to 1 mL, then 10 µL solutions were adsorbed on formvar/ carbon coated grids and stained with 2% aqueous uranyl acetate. Afterward, the grid was dried in air for 1 h at room temperature and observed using TEM.

### 5.9 Environmental Scanning Electron Microscope

The microstructure and morphology of the samples were characterized using an Environmental Scanning Electron Microscope (E‐SEM, ThermoFisher, Quanta 200 FEG). The system is equipped with a Schottky field‐emission gun electron source, operating at 30 kV accelerating voltage and beam currents up to 100 nA, and offers three vacuum modes—high vacuum, low vacuum, and environmental/wet modes; we used the environmental mode for our samples to maintain the integrity of the liquid phase during imaging. For liquid sample preparation, a 10 µL aliquot of the solution was carefully adsorbed onto a formvar/ carbon coated grid. The grid was allowed to dry under ambient conditions for 2 min to ensure proper adsorption of the sample.

### 5.10 Confocal Microscopy of Microdroplets With BDPg in the Core and DiI in the Lipid Shell

To visually confirm payload localization, microdroplets were formulated using a microfluidic system that included the Herringbone Mixer Glass Chip at aqueous‐to‐organic phase flow rates of 150:50 µL/min. The conjugation of DiI to DSPE‐PEG2k was done according to a previous study [80] with a mass ratio of 1.9 to 100 (DiI to lipids: DSPC and DSPE‐PEG2k) in PBS: glycerol: propylene glycol mixture. The aqueous phase contained PBS, glycerol, and propylene glycol (8:1:1 v/v), along with DSPC:DSPE‐PEG2k (9:1 molar ratio, 2.5 mg/mL) with DiI. The organic phase contained PFH (10 µL/mL) and BDPg (0.1 mg/mL) dissolved in ethanol. Microdroplets were collected and imaged using a Leica SP8 confocal microscope (63 × /1.40 oil immersion objective, zoom ×1 and ×5). BDPg (green) was excited using an argon laser, with emission collected at 485–513 nm (PMT1). DiI (red) was excited using a DPSS 561 nm laser, with emission collected at 543–573 nm (PMT3). Images were acquired and processed using LAS X software.

### 5.11 In Vitro Evaluation of NDs in 4T1 Breast Cancer Cells

In vitro studies were conducted using 4T1 cells, a metastatic triple‐negative murine breast carcinoma cell line (purchased from ATCC), to evaluate the therapeutic potential of C_6_F_14_ ND‐mediated mechanotherapy. The cells were cultured in RPMI 1640 Medium (With ʟ‐glutamine, supplemented with 10% v/v fetal bovine serum, 1% v/v penicillin‐streptomycin) at 37°C in a humidified 5% CO_2_ incubator. On the day of the experiment, cells (≈85% confluency) were dissociated with TrypLE Express (Gibco Corp, 12604‐013, Grand Island, NY, USA) and resuspended at 1 × 10^6^ cells/mL in degassed PBS containing calcium and magnesium (PBS+/+).

Prior to US experiments, we conducted a concentration optimization study to determine the non‐cytotoxic range of C_6_F_14_ NDs (blank NDs) for 4T1 breast cancer cells. Cells were seeded in 6‐well plates at a density of 2 × 10^5^ cells per well in 2 mL RPMI 1640 Medium (With ʟ‐glutamine, supplemented with 10% v/v fetal bovine serum, 2.5% v/v penicillin‐streptomycin) and exposed to increasing volumes of NDs (25–200 µL of 2.52 × 10^11^ particles/mL suspension, with three replicates each), with medium replacement at 48 h to maintain nutrient availability and remove non‐adherent cells. After 72 h of exposure, cells were collected in TrypLE Express, and viability was quantified via direct cell counting and normalized to non‐treated controls (NTC) to account for baseline proliferation. This screening identified the optimal ND volume that maintained >90% viability while ensuring high ND concentration for therapeutic experiments. All treatments were analyzed in triplicate.

For the US‐mediated therapy experiments, 4T1 cells were prepared at a concentration of 1 × 10^6^ cells/mL in PBS containing calcium and magnesium. Aliquots of 200 µL (containing 2 × 10^5^ cells) were distributed into 0.5 mL Eppendorf tubes and divided into six experimental groups with six replicates each. The treatment groups included NTC, US alone (combined 105 kHz and 3.5 MHz transducers), NDs alone at the predetermined optimal concentration (1.26 × 10^10^ NDs), and three combination groups pairing NDs with either an imaging US transducer, therapeutic US transducer, or dual‐frequency system containing both. Hydrophone calibration inside the 0.5 mL Eppendorf tubes showed a 30% acoustic PNP attenuation, consistent with literature findings for similar setups [43]. Each treated sample was positioned at the focal point of our custom US system, featuring a rotating imaging transducer (3.5 MHz, MI 1.67) and a therapeutic transducer (105 kHz, MI 0.84), each operating for 120 s. The therapeutic transducer delivered pulsed waves with 0.5 ms burst length and 30 ms period. After treatment, cells were transferred to six‐well tissue culture dishes containing RMPI 1640 medium supplemented with 2.5% v/v penicillin‐streptomycin and cultured at 37°C in a humidified 5% CO_2_ incubator for 72 h, with medium replacement at 48 h. Live cells were counted after TrypLE Express dissociation. All experiments were performed in triplicate.

### 5.12 Combined Mechanical‐Chemotherapeutic Efficacy of 5‐FU Loaded NDs

In vitro studies of 5‐FU NDs were conducted using breast cancer cells (4T1 with stable lentiviral expression of the ZsGreen1 protein for confocal microscopy, or regular 4T1 cells) to evaluate the therapeutic potential of US‐mediated mechanotherapy and drug delivery of 5‐FU. The cells were cultured in RPMI 1640 Medium (With ʟ‐glutamine, supplemented with 10% v/v fetal bovine serum, 1% v/v penicillin‐streptomycin) at 37°C in a humidified 5% CO_2_ incubator. On the day of the experiment, cells (≈85% confluency) were dissociated with TrypLE Express (Gibco Corp, 12604‐013, Grand Island, NY, USA) and resuspended at 1 × 10^6^ cells/mL in degassed PBS containing calcium and magnesium (PBS+/+).

For the experiments, 4T1 cells were prepared at a concentration of 1 × 10^6^ cells/mL in PBS containing calcium and magnesium. Aliquots of 200 µL (containing 2 × 10^5^ cells) were distributed into 0.5 mL Eppendorf tubes. NDs (blank NDs or 5‐FU NDs) at the predetermined optimal concentration from the previous experiment (1.26 × 10^10^ NDs), free 5‐FU, and 5‐FU NDs at the same drug concentration based on 5‐FU ND formation (25 µg/mL ND solution). For the first experiment (*n* = 6 per experimental group), containing regular 4T1 cells, the groups included NTC (group 1), blank NDs without US (group 2), 5‐FU NDs without US (group 3), free 5‐FU (group 4), blank NDs + dual‐frequency system (group 5), and 5‐FU NDs + dual‐frequency system (group 6). Each treated sample was positioned at the focal point of our custom US system, featuring a rotating imaging transducer (3.5 MHz, MI 1.67) and a therapeutic transducer (105 kHz, MI 0.84), each operating for 120 s. The therapeutic transducer delivered pulsed waves with a 0.5 ms burst length and 30 ms period, ensuring synchronized ND vaporization (imaging), cavitation, and drug release (therapeutic) effects. After treatment, cells were transferred to six‐well tissue culture dishes containing RMPI 1640 medium supplemented with 2.5% v/v penicillin‐streptomycin and cultured at 37°C in a humidified 5% CO_2_ incubator for 72 h, with medium replacement at 48 h. Live cells were counted after TrypLE Express dissociation.

For the second experiment (*n* = 3 per experimental group), GFP‐expressing 4T1 breast cancer cells (4T1 with stable lentiviral expression of the ZsGreen1 protein) were used, divided into four groups: NTC (group 1), 5‐FU NDs (1.26 × 10^10^ NDs) co‐incubated with 4T1 cells without US exposure to assess passive drug release (group 2), 5‐FU NDs (1.26 × 10^10^ NDs) exposed to dual‐frequency US in Eppendorf tubes without cells, followed by addition of the resulting chemotherapy solution to 4T1 cell culture to isolate the chemotherapy effect (group 3), and 5‐FU NDs (1.26 × 10^10^ NDs) combined with 4T1 cells subjected to simultaneous dual‐frequency US application, representing mechanotherapy plus drug release (group 4). Each treated sample was positioned at the focal point of a custom US system comprising a rotating imaging transducer (3.5 MHz, MI 1.67) and a therapeutic transducer (105 kHz, MI 0.84), each operating for 120 s. The therapeutic transducer delivered pulsed waves with a 0.5 ms burst length and 30 ms period. Post‐treatment, cells were transferred into six‐well tissue culture plates containing RPMI 1640 medium supplemented with 2.5% penicillin–streptomycin and incubated at 37°C in a humidified atmosphere with 5% CO_2_ for 72 h, with medium replaced at 48 h.

Prior to confocal microscopy, fresh medium was supplied, and imaging was performed on an Olympus FV3000‐IX83 system (Olympus, Japan) equipped with a UPLXAPO 20 × objective (NA 0.7). GFP fluorescence was excited using a 488 nm laser (1.75% transmissivity at 100% power) and detected through the Alexa Fluor 488 channel. Paired brightfield and fluorescence images (512 × 512 pixels, 12‐bit depth) were captured at a pixel size of 1.24 µm (0.6215 µm/pixel with 2 × digital zoom) using a Galvano scanner in line sequential mode at 2.114 µs/pixel. Untreated GFP‐expressing 4T1 cells served as imaging controls. All image processing was performed using ImageJ (NIH).

### 5.13 Fluorescence Characterization of NDs by Multispectral IVIS Imaging

The fluorescence profiles of BDPg ND, BDPr ND, and BDPfr ND were characterized using an IVIS imaging system. Five Eppendorf tubes were arranged (left to right: PBS control, BDPg ND, BDPr ND, BDPfr ND, and air blank) and imaged across 15 excitation/emission filter pairs spanning visible to near‐infrared wavelengths (480–740 nm excitation steps of 20 nm, 620/710/790 nm emission). This multispectral approach enabled resolution of ND‐specific fluorescence signals while accounting for potential background autofluorescence. All acquisitions used identical instrument settings to permit direct comparison between formulations.

### 5.14 Mice Breast Cancer Model and Treatment Protocol

All animal procedures were approved by the Institutional Animal Care and Use Committee (IACUC) of Tel Aviv University and were carried out in compliance with institutional guidelines for care and use of animal models (approval TAU‐MD‐IL‐2506‐132‐4). MET‐1 [81] mouse breast carcinoma cells were injected into 8 to 12 weeks old female FVB/NHanHsd mice (Envigo, Jerusalem, Israel). The cells were cultured at 37°C in a humidified 5% CO_2_ incubator in Dulbecco's modified Eagle medium (DMEM, high glucose, supplemented with 10% v/v fetal bovine serum, 1% v/v penicillin‐streptomycin, and 0.11 g L^−1^ sodium pyruvate) until ≈85% confluency, then dissociated with TrypLE Express and resuspended in PBS+/+. During the day of the injection, Met‐1 cells were collected with TrypLE Express dissociation reagent to a final concentration of 1 × 10^6^ cells in 25 µL of PBS+/+. The cells were subcutaneously injected into #4 inguinal mammary fat pads to establish tumors. The tumor size was recorded every 4 days until they reached approximately a volume of 80–100 mm^3^, unless otherwise specified for individual experiments. Tumor volume was estimated using the ellipsoid approximation (equation 2):

(2) $$\text{Tumor}\;\text{volume}=\mathrm{LxWx}\mathrm{Hx}\frac{\pi }{6}$$

where L, W, and H are the tumor length, width, and height, respectively, measured with a caliper. Mice were anesthetized with isoflurane (Piramal), the fur in the tumor area was shaved and depilated (Veet hair removal cream), and positioned within the US transducer focal zone for treatment.

### 5.15 In Vivo Imaging and Pharmacokinetic Analysis

Biodistribution, tumor accumulation, and blood circulation profiles of BDPfr NDs vs. free dye were characterized to establish clearance kinetics, organ distribution patterns, and tumor targeting efficiency prior to therapeutic studies

The mice were divided into 3 groups: NTC (*n* = 3), free BDPfr (*n* = 4), and BDPfr NDs (*n* = 4) injection. 150 uL of diluted BDPfr NDs (150 µL total volume: 50 µL ND solution + 100 µL PBS(−/−)) or free BDPfr (150 µL at equivalent dye concentration (0.2 µm BDPfr) in 5% ethanol/PBS) were injected retroorbitally when the tumor volume reached an average of 1000 mm^3^, the mice were anesthetized using isoflurane. Fluorescence levels were assessed and analyzed with 640 nm excitation and 710 nm emission for whole mice at different time points during 24 h and different organs (tumor, liver, spleen, kidneys and brain) ex vivo 24 h post injection using IVIS SpectrumCT (Caliper IVIS Lumina Series III) in vivo imaging system and Living Image 4.8.2 software (PerkinElmer, USA) using background subtraction and radiant efficiency quantification ([photons/s/cm^2^/sr]/[µW/cm^2^]).

In a separate pharmacokinetic experiment, the blood biodistribution and circulation time of BDPfr NDs were evaluated. Animals were assigned to either NTC (*n* = 3) or BDPfr ND (*n* = 3) groups. Once tumors reached a volume of approximately 1000 mm^3^, BDPfr NDs were administered via retro‐orbital injection under isoflurane anesthesia (Piramal, Israel). The BDPfr ND group received 150 uL per 20 g mouse (50 uL ND solution + 100 uL PBS(−/−)), while the NTC group provided baseline fluorescence measurements. Serial blood samples were collected via cheek pouch puncture at 10 min, 3 h, and 24 h post‐injection following a staggered sampling schedule adapted from established protocols to minimize animal stress [82]. Blood was immediately transferred into 96‐well plates, and fluorescence was quantified ex vivo using an IVIS Spectrum/SpectrumCT system (Caliper IVIS Lumina Series III, PerkinElmer, USA) with 640 nm excitation and 710 nm emission. Radiant efficiency ([photons/s/cm^2^/sr]/[µW/cm^2^]) was calculated by defining regions of interest (ROIs) in Living Image 4.8.2 software (PerkinElmer), enabling comparative pharmacokinetic analysis between BDPfr ND treated and NTC groups.

### 5.16 Tissue Harvesting and Histology Protocol

For ex vivo therapeutic assessment, tumors were harvested. In addition, after the ex vivo biodistribution study using IVIS, tumors and internal organs (spleen, liver, kidneys, and brain) were harvested. Tumors and internal organs were frozen using liquid nitrogen and methyl butane and processed for embedding in O.C.T Compound (ThermoFisher Scientific, USA), and then stored at −80°C. Frozen samples were subsequently cryo‐sectioned into 12 µm thick slices in a −20°C cryostat microtome (CM1950, Leica Biosystems), and processed via two parallel pathways: (1) for histological evaluation of US treatment, sections were stained with hematoxylin (Leica 3801542) and eosin (Leica 3801602) (H&E) following standardized protocols [83] and stored at −80°C, (2) for fluorescent ND biodistribution analysis, sections were mounted on glass slides and treated with WGA Alexa Fluor 488, washed with PBS and then treated with three drops of DAPI containing mounting medium (Fluoroshield, ab104139, Abcam), then coverslipped and allowed to develop for 15 min at room temperature in light protected conditions and stored at −80°C before further analysis.

### 5.17 In Vivo Mechanotherapy in a Breast Cancer Mouse Model

Blank ND‐mediated mechanical tissue fractionation was evaluated using the dual‐frequency US system prior to combined therapy studies. Mice were randomized into four groups (*n* = 3/group): (1) NTC, (2) NDs + imaging US (3.5 MHz, MI 1.84), (3) NDs + therapeutic US (105 kHz, MI 0.91), and (4) NDs + combined dual‐frequency US. Anesthetized mice received retro‐orbital injection of blank NDs (110 µL) and were positioned atop the water tank using an agarose spacer to place tumors at the dual‐frequency focal zone after 10 min circulation. US was administered for 6 min (2‐min sonications per plane across three *z*‐axis planes spaced by 2.5 mm to cover the tumor volume, given the imaging transducer's 2.4 mm focal spot). Combined transducer groups received simultaneous imaging/therapeutic US. Mice were sacrificed at 24 h post‐treatment; tumors were frozen in liquid nitrogen‐cooled methylbutane (−80°C storage) and sectioned (12 µm, Leica CM1950 cryostat at −20°C) for H&E staining to assess tissue fractionation.

### 5.18 Microscopy Imaging and Quantitative Analysis

Following thawing to room temperature, tissue slide sections were imaged using the Echo Revolution microscope (Echo, San Diego, USA). Scans were performed at 4 × and 20 × optical magnification to assess both tumor fractionation morphology in H&E‐stained sections (using the brightfield channel) and organ‐specific ND extravasation patterns in fluorescent samples. For fluorescence imaging, optimized excitation wavelengths were applied: 365 nm (DAPI), 410 nm (GFP), 550 nm (TRITC), and 690 nm (Cy5), allowing simultaneous visualization of cellular architecture and ND distribution. This imaging protocol facilitated correlative analysis of therapeutic effects at the tissue level and drug delivery efficiency at the cellular scale. For comparison and quantification of the resulting lesions, post‐processing of the scanned images was performed in ImageJ. Each image was converted to a binary image, where the lesion areas were turned black, and non‐lesion areas were white. The lesion area percentage was calculated using the following Equation (3):

(3) $$\mathrm{Lesion}\;\mathrm{area}\;[\%]=\frac{\mathrm{Number}\;\mathrm{of}\;\mathrm{black}\;\mathrm{pixels}}{\mathrm{Number}\;\mathrm{of}\;\mathrm{total}\;\mathrm{pixels}}*100$$

To assess intra‐tumoral distribution of BDPfr NDs, tumors were harvested 24 h post injection and processed for embedding in O.C.T Compound (ThermoFisher Scientific, USA). The O.C.T blocks were cut into 12 µm sections, which were made serially through multiple regions of each tumor. Slides were then examined and imaged by Echo Revolution microscope (Echo, San Diego, USA) Then, tumor slides were randomly chosen and underwent WGA Alexa Fluor 488 (cell membrane), PBS washing, then DAPI immunomounting for cell nuclei, as mentioned in the tissue harvesting protocol section. Dried slides were examined and imaged by a TCS SP8 multiphoton confocal microscope (Leica, USA).

### 5.19 Survival Study

The long‐term therapeutic effect of combined mechanotherapy and chemotherapy release was evaluated in bearing MET‐1 breast carcinomas, with each mouse having a single tumor. Four groups were included in this study: 1) NTC group, 2) a group that received 5‐FU ND treatment alone without US (110 µL 5‐FU ND, 1.25 µg 5‐FU), 3) free 5‐FU treatment (1.25 µg 5‐FU in 110 µL PBS(−/−)), 4) Blank ND‐mediated histotripsy (110 µL Blank ND + US) and 5) 5‐FU ND‐mediated histotripsy (110 µL 5‐FU ND + US, 1.25 µg 5‐FU) (*n* = 8 mice per group). US treatment was done with a dual‐frequency system (3.5 MHz, MI 1.84, and 105 kHz, MI 0.91). Treatments were repeated weekly, and tumor growth was monitored twice a week. The mice were sacrificed when the tumor volume reached 1000 mm^3^.

In a separate endpoint imaging experiment, a subset of mice from the NTC, Blank ND + US, and 5‐FU ND + US groups received an injection of BDPfr NDs when tumor volume reached 1,000 mm^3^. Tumors and organs were harvested and scanned ex vivo by IVIS 4 h post‐injection. The imaging cohort consisted of four groups (*n* = 3 per group): control (no BDPfr NDs), BDPfr NDs only, BDPfr NDs after Blank ND + US treatment, and BDPfr NDs after 5‐FU ND + US treatment.

### 5.20 Statistical Analysis

Prism 10.1.2 (GraphPad Software) was employed for statistical analysis. In experiments involving multiple groups, differences among multiple populations and subpopulations were assessed using One‐Way ANOVA with Tukey's multiple comparisons. The value of p ≤ 0.05 was considered statistically significant. Differences are presented on graphs using the following abbreviations: blank. for not significant, ^*^ for *p* ≤ 0.05, ^**^ for *p* ≤ 0.01, ^***^ for *p* ≤ 0.001, and ^****^ for *p* ≤ 0.0001.

### 5.21 Declaration of Generative AI Use

Generative AI tools (ChatGPT (OpenAI)) were used to assist with language editing and manuscript preparation. The authors reviewed and take full responsibility for all content.

## Author Contributions

**T.B**. designed and performed the research, analyzed the data, and wrote the manuscript. **M.B., and M.G**. assisted with in vivo experiments. **D.P**. guided, advised, and contributed to the manuscript. **T.I**. guided, advised, designed the research, and contributed to the manuscript. All authors reviewed the manuscript.

## Funding

This work was supported in part by the Israel Science Foundation under Grant 192/22, in part by an ERC StG under Grant 101041118 (NanoBubbleBrain), in part by the Israel Cancer Research Fund (grant number 1286686), in part by the Nicholas and Elizabeth Slezak Super Center for Cardiac Research and Biomedical Engineering at Tel Aviv University, in part by a Zimin Institute grant, and in part by the Marian Gertner Institute for Medical Nanosystems and The Cancer Biology Research Center (CBRC) at Tel Aviv University (T.B.).

## Ethics Statement

All animal procedures were approved by the Institutional Animal Care and Use Committee (IACUC) of Tel Aviv University and were carried out in compliance with institutional guidelines for care and use of animal models (approval TAU‐MD‐IL‐2506‐132‐4).

## Conflicts of Interest

D.P. receives licensing fees (to patents on which he was an inventor) from, invested in, consults (or on scientific advisory boards or boards of directors) for, lectured (and received a fee), or conducts sponsored research at TAU for the following entities: ART Biosciences, BioNtech SE, Earli Inc., Geneditor Biologics Inc., Kernal Biologics, Newphase Ltd., NeoVac Ltd., RiboX Therapeutics, SirTLabs Corporation, Teva Pharmaceuticals Inc. All other authors declare no competing financial interests.

## Supporting information




**Supporting File**: advs78010‐sup‐0001‐SuppMat.pdf.

## Data Availability

The datasets generated during and/or analyzed during the current study will be made available upon request.
