# Supplementary material for: Acoustically Activatable Drug‐Loaded Nanodroplets for Mechanochemical Therapy in Solid Tumors
Source: Adv Sci (Weinh). 2026 Sep 27:e78010. Online ahead of print. doi: 10.1002/advs.78010 (PMC13616419; doi:10.1002/advs.78010)
Supplement: Supplementary file 1 [file ADVS-9999-e78010-s001.pdf]

Supporting information for

Acoustically activatable drug-loaded nanodroplets for mechanochemical therapy in solid tumors

Tiran Bercovici[1], Mike Bismuth[1], Meir Goldsmith[2,3,4,5], Dan Peer[2,3,4,5], Tali Ilovitsh[1,5,6*]

[1] School of Biomedical Engineering, Tel Aviv University, Tel Aviv, 69978 Israel
[2] Laboratory of Precision NanoMedicine, The Shmunis School of Biomedicine and Cancer Research, George S. Wise Faculty of Life Sciences, Tel Aviv University, Tel Aviv, 69978 Israel
[3] Department of Materials Sciences and Engineering, Iby and Aladar Fleischman Faculty of Engineering, Tel Aviv University, Tel Aviv, 69978 Israel
[4] Cancer Biology Research Center, Tel Aviv University, Tel Aviv, 69978 Israel
[5] Center for Nanoscience and Nanotechnology, Tel Aviv University, Tel Aviv, 69978 Israel
[6] The Sagol School of Neuroscience, Tel Aviv University, Tel Aviv, 69978 Israel
* Corresponding author: ilovitsh@tauex.tau.ac.il

This PDF file includes:
Supplementary Tables
Table S1

Supplementary Figures
Fig. S1-S12

Table S1 - Physical characteristics of PFC and other materials useful for making blank encapsulated NDs.

Compound	Molecular Weight (g/mol)	Boiling Point (°C)	Phase at RT	Solubility in Water	Excitation/ Emission Wavelengths (nm)
Perfluoropentane (C_5F_{12})	288.05	29.5	Liquid	Insoluble	NA
Perfluorohexane (C_6F_{14})	338.06	56	Liquid	Insoluble	NA
5-Fluorouracil (5-FU)	130.08	Sublimes at 282-283	Solid	Highly Soluble in organic solvents, low solubility in water.	Absorption: 265
BODIPY 493/503	262.11	NA	Solid	Soluble in organic solvents, Insoluble in water.	Ex: 493, Em: 503
BDP TR-X-NHS ester	634.46	NA	Solid	Soluble in DMSO, DMF. Insoluble in water.	Ex: 589, Em: 616
BDP® 630/650-X-NHS ester	660.5	NA	Solid	Soluble in DMSO, DMF. Insoluble in water.	Ex: 628, Em: 642

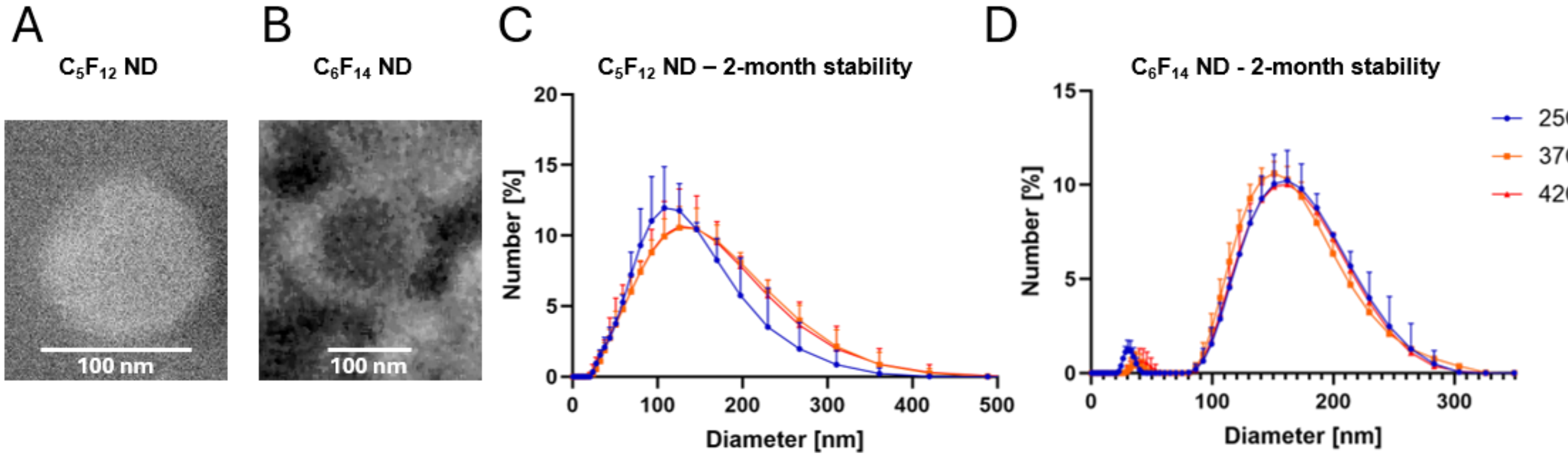

Figure S1. Structural, and size stability of perfluorocarbon ND. (A) ESEM image of C_5F_{12} NanoAssemblr-formulated ND (scale bar: 100 nm). (B) ESEM image of C_6F_{14} NanoAssemblr-formulated ND (scale bar: 100 nm). (C) Number-weighted DLS size distributions of C_5F_{12} NanoAssemblr ND after 2 months storage at 2-8 °C, measured at 25, 37, and 42 °C. (D) Number-weighted DLS size distributions of C_6F_{14} NanoAssemblr ND after 2 months storage at 2-8 °C, measured at 25, 37, and 42 °C.

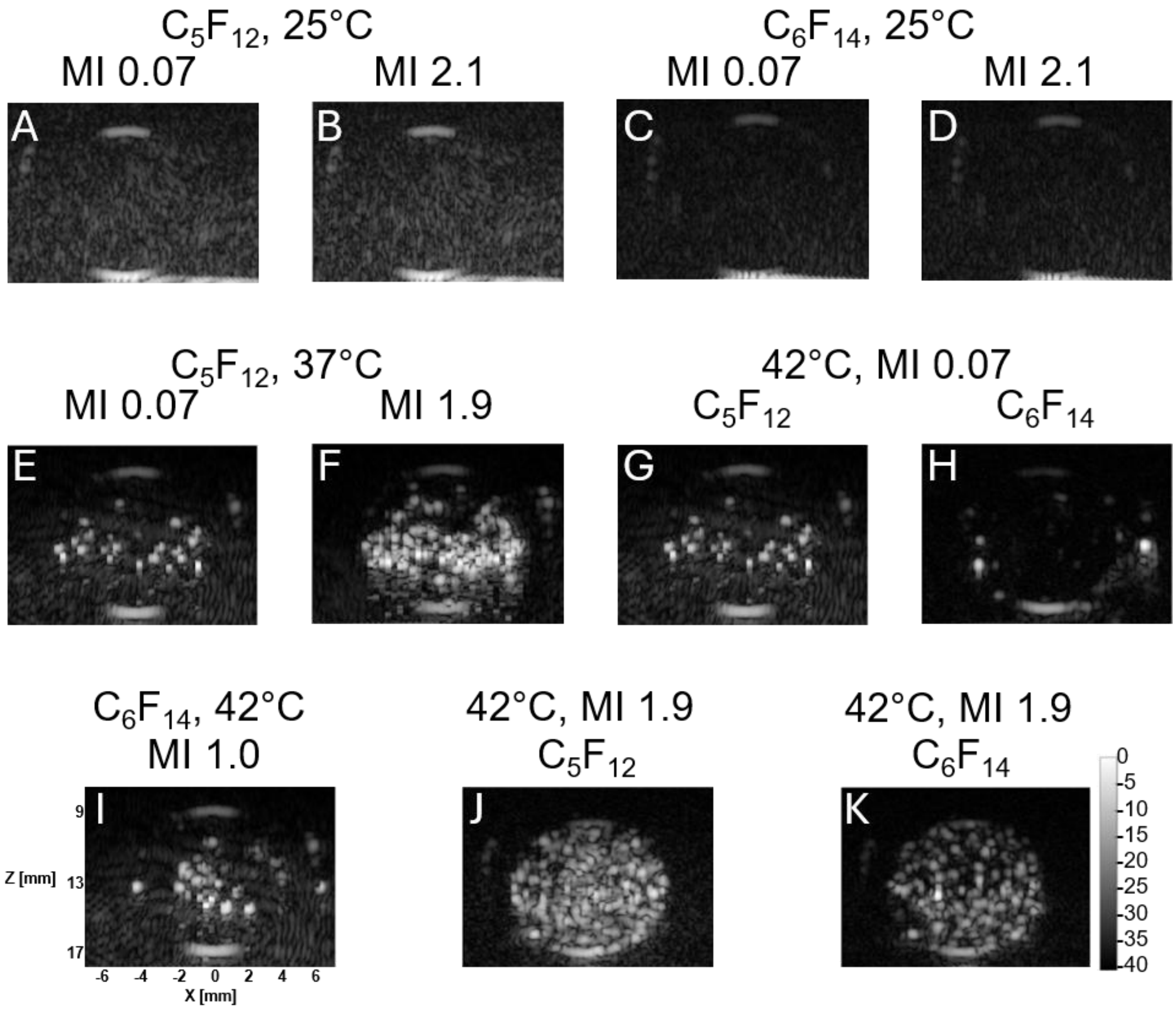

Figure S2. Acoustic imaging of ADV in C_5F_{12} and C_6F_{14} NDs fabricated at 12 mL/min under varying temperature and MI. At 25 °C, neither formulation showed ADV even at MI 2.1, confirming thermal stability at ambient temperature (A-D). At 37 °C, C_5F_{12} NDs showed no ADV at MI 0.07 but pronounced ADV at MI 1.9, demonstrating pressure-dependent phase transition (E,F). At 42 °C, both formulations showed minimal ADV at MI 0.07, with partial ADV in C_6F_{14} NDs at MI 1.0 and enhanced ADV in both at MI 1.9, reflecting combined thermal and acoustic triggering (G-K). Scale bar is common to all subfigures.

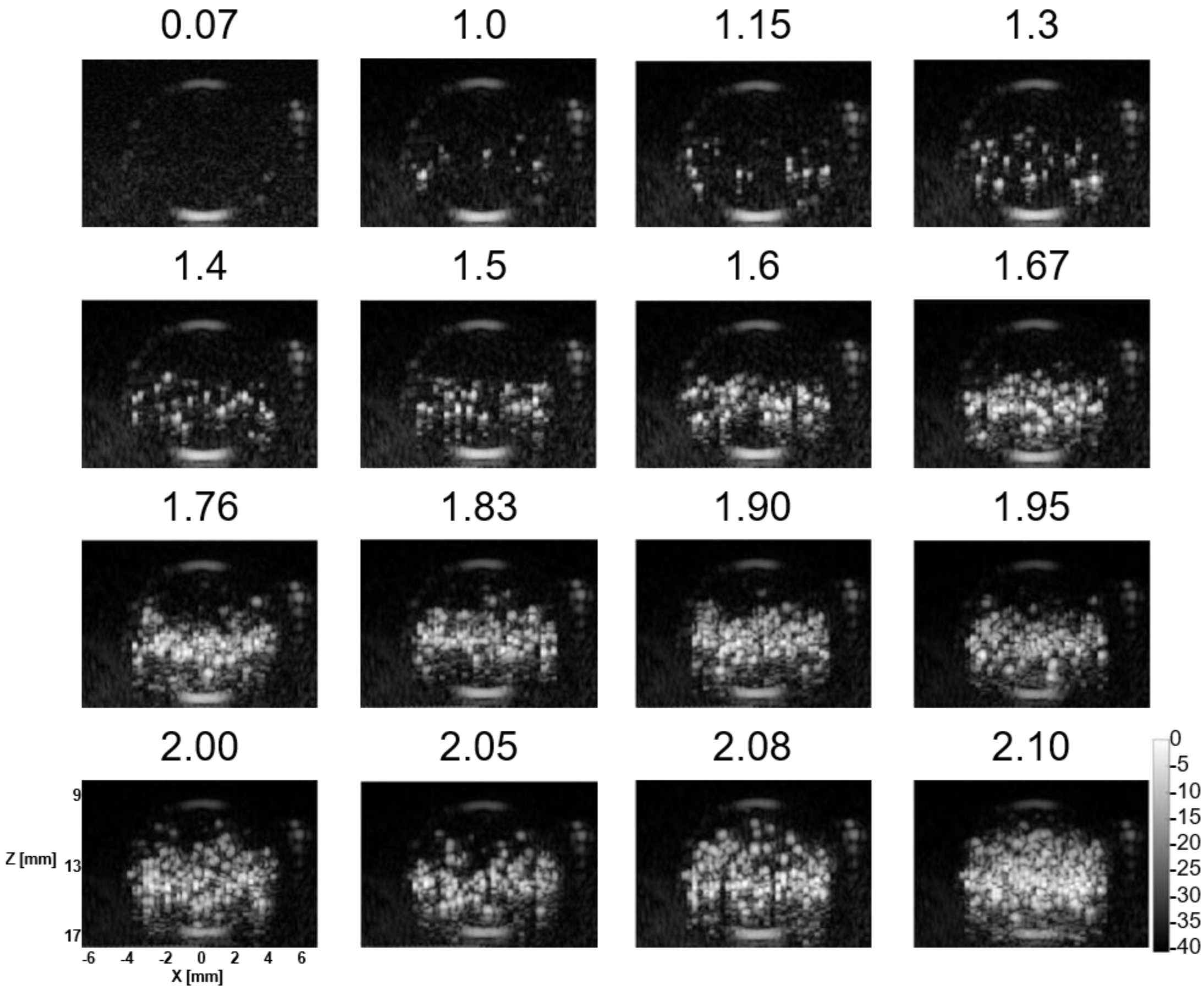

Figure S3. Acoustic imaging of C_6F_{14} NanoAssemblr ND ADV at 37 °C under varying MIs. Images were captured at increasing MIs (0.07-2.10). At low MI (0.07), no ADV was observed. Sparse MBs begin to appear at MI ≈ 1.0, with progressive vaporization and MB accumulation as MI increases (1. 0-2.1). Pronounced ADV occurs at MI ≥ 1.67, reaching maximal MB generation near the highest tested MI (2.1). The results demonstrate the pressure-dependent nature of ND ADV and confirm that efficient phase transition occurs near the clinical safety limit (MI = 1.9). Scale bar is common to all subfigures.

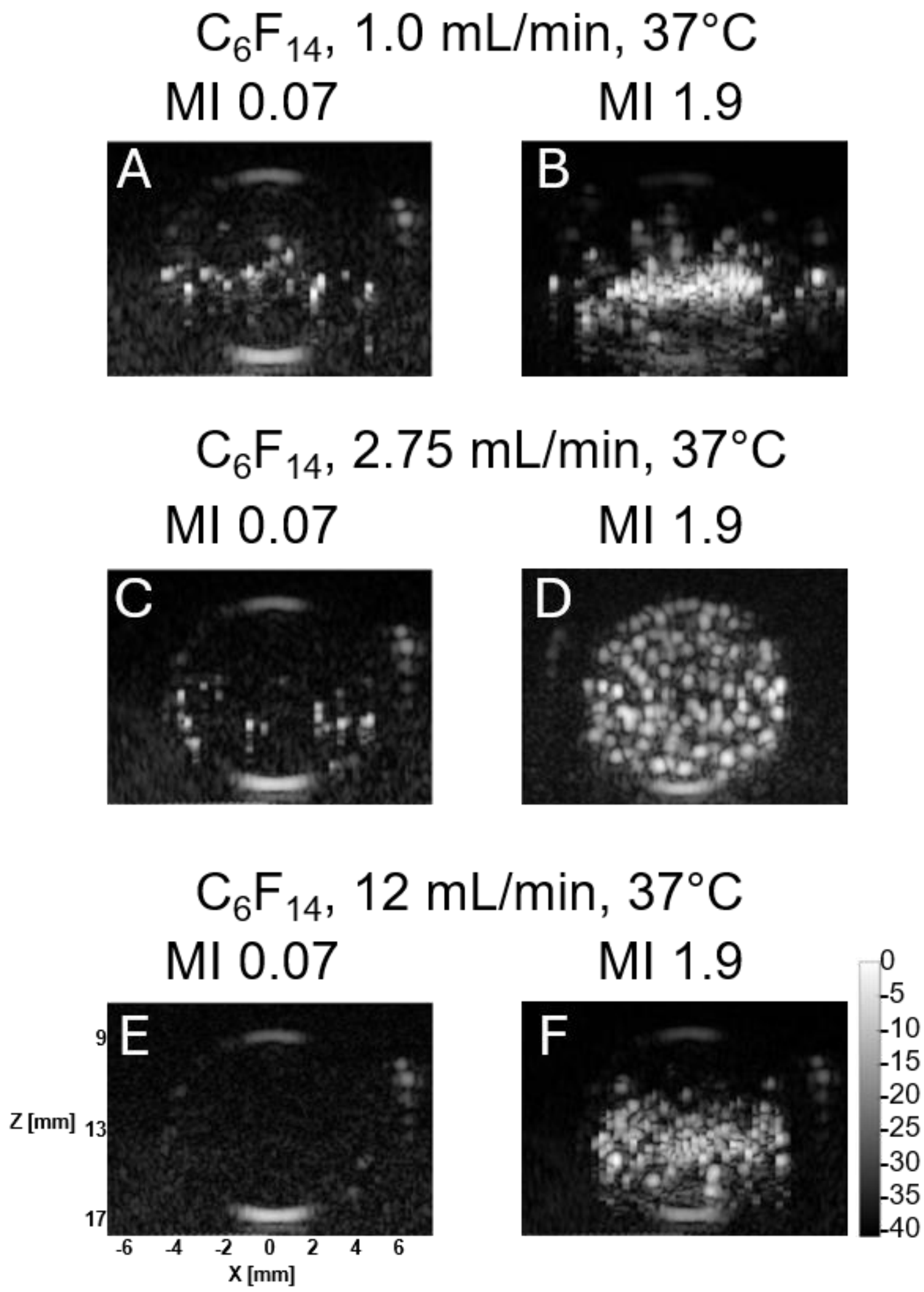

Figure S4. Effect of microfluidic TFR on ADV behavior of C_6F_{14} NDs at 37 °C. Acoustic images showing pressure-dependent ADV of C_6F_{14} NDs synthesized at different microfluidic TFRs. (A) NDs prepared at TFR = 1.0 mL/min show spontaneous vaporization and unstable MB formation at MI 0.07, while (B) pronounced ADV is detected at MI 1.9. (C) NDs prepared at TFR = 2.75 mL/min show limited spontaneous ADV at MI 0.07 but remain more stable than the lower-TFR batch, while (D) strong ADV is observed at MI 1.9. (E) NDs prepared at TFR = 12 mL/min show no visible vaporization at MI 0.07, while (F) robust ADV is observed at MI 1.9. Across all TFRs, MI 1.9 produced stronger ADV than MI 0.07. Overall, increasing TFR yields smaller, acoustically more stable NDs that require higher pressure for vaporization. Scale bar is common to all subfigures.

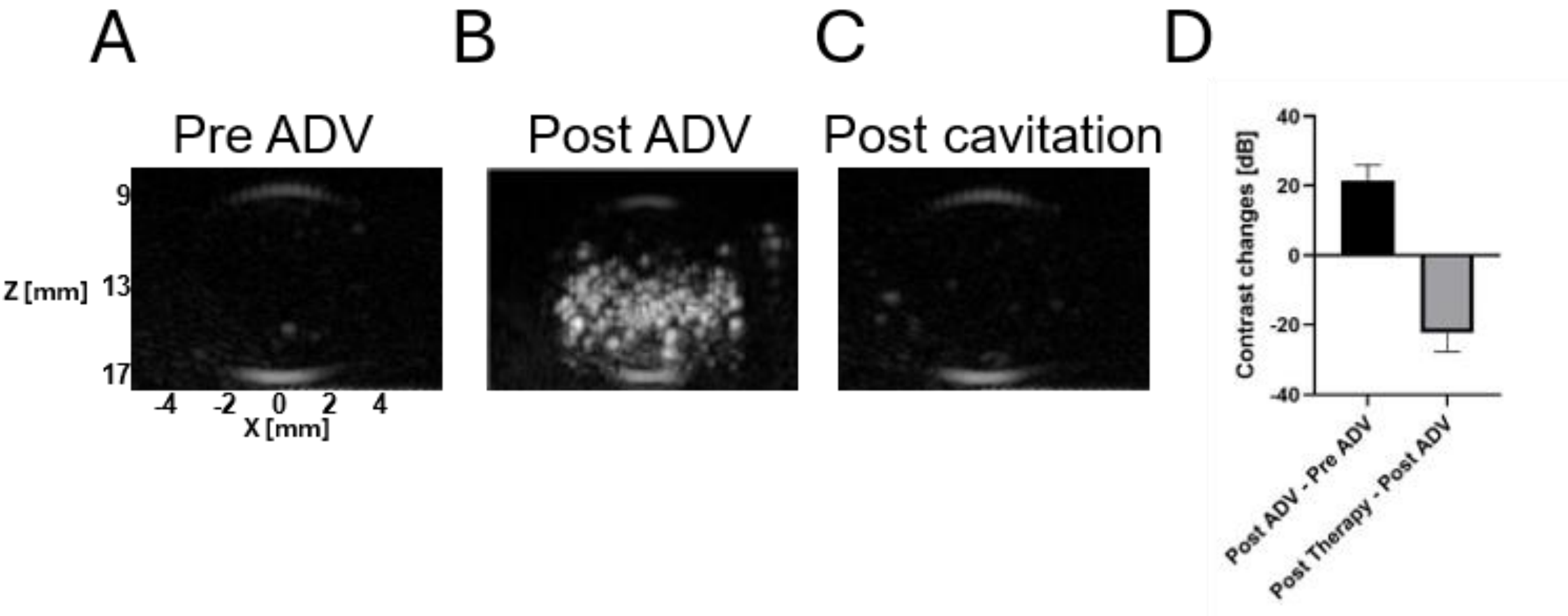

Figure S5. Characterization of contrast loss following low-frequency cavitation of vaporized NDs. (A) B-mode US images of NDs before ADV. (B) ADV of C_6F_{14} NDs at MI 1.9 (5.5 MHz). (C) Post-cavitation of activated MBs (MI 0.84, 105 kHz). (D) Quantification of contrast changes following ADV and cavitation. Contrast increased after ADV, confirming ND vaporization, and decreased after cavitation, indicating destruction of the vaporized NDs. Data are presented as mean ± SD, n = 3.

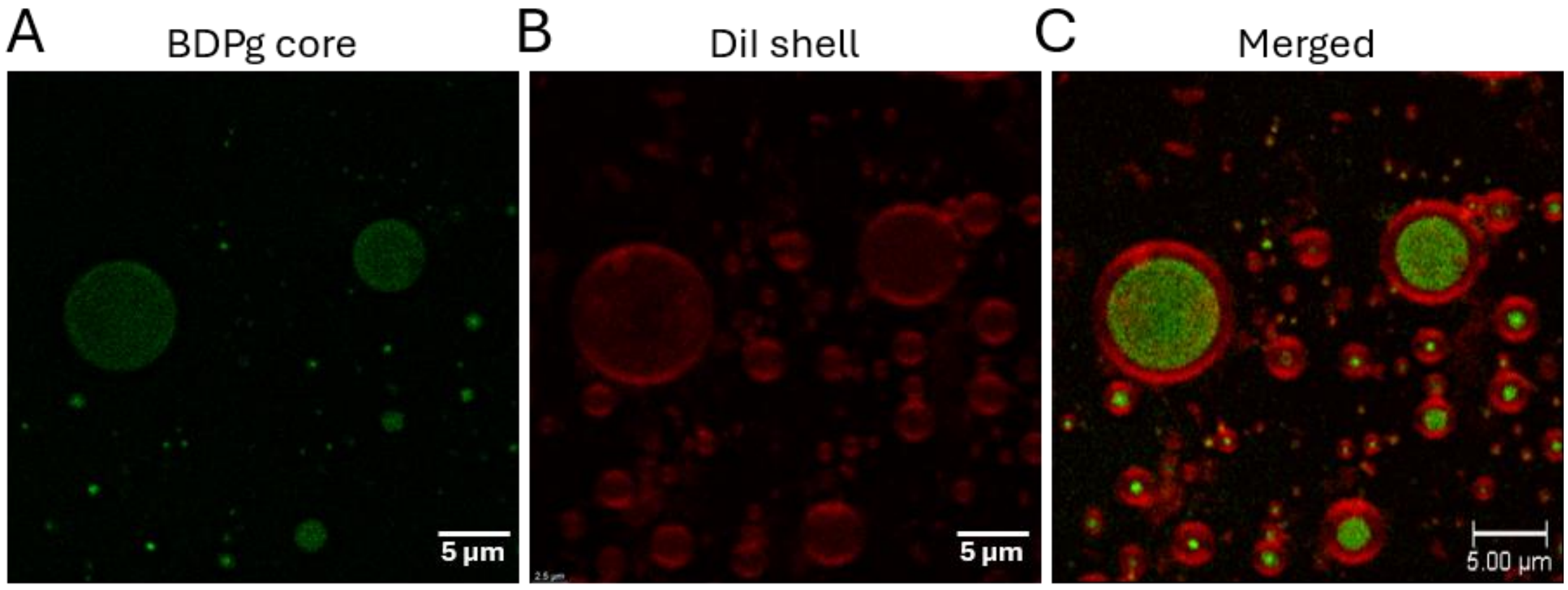

Figure S6. Confocal fluorescence images of microdroplets containing BDPg in the PFC core (green) and DiI tagged lipid shell (red). (A) Green BDPg channel. (B) Red DiI channel. (C) Merged image. Scale bar: 5 µm.

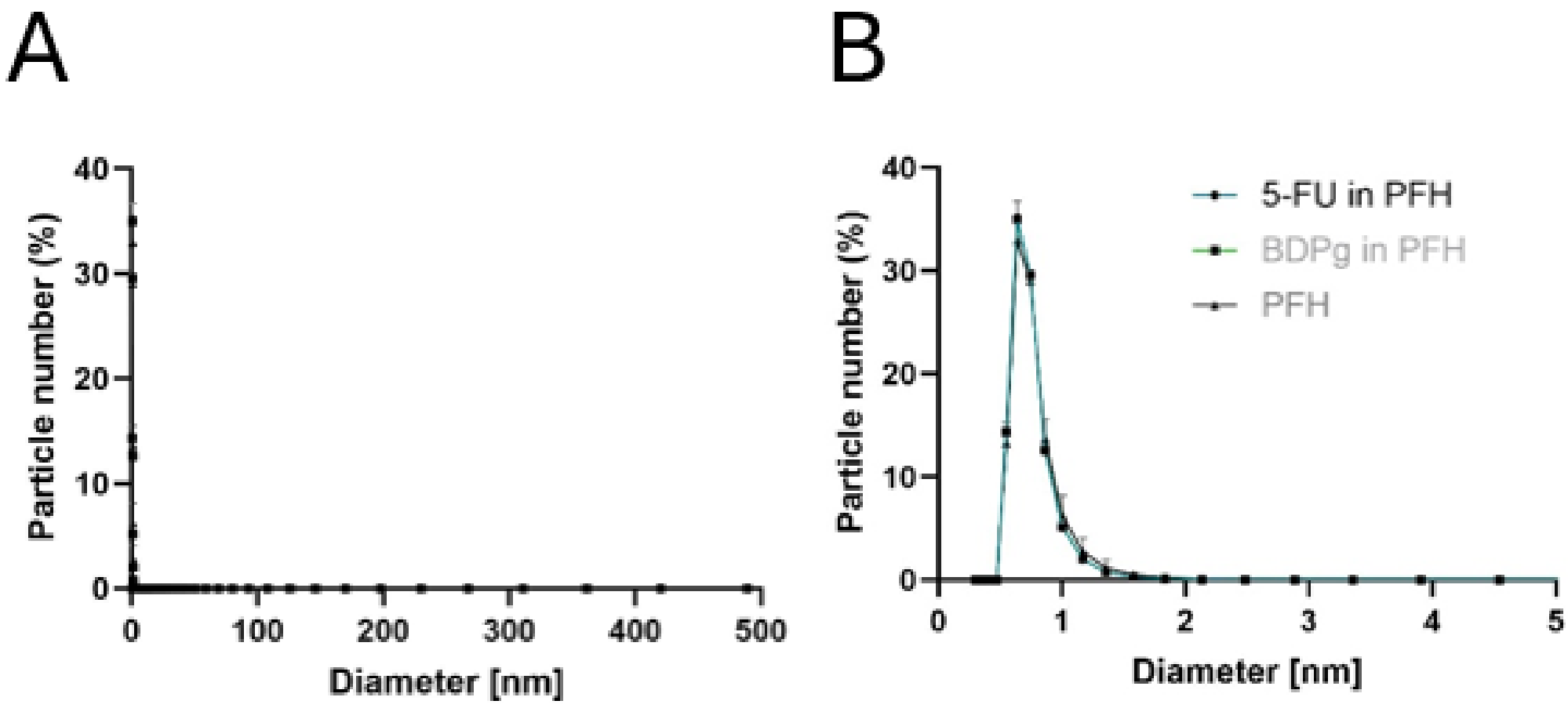

Figure S7. DLS size distribution of pure PFH, BDPg dissolved in PFH (0.1 mg/mL), and 5-FU dissolved in PFH (0.1 mg/mL). (A) Full range (0-500 nm diameter) showing no large aggregates or secondary populations. (B) Zoomed range (0-5 nm). All samples exhibited hydrodynamic diameters below 1 nm (PFH: 0.95 ± 0.08 nm; BDPg/PFH: 0.96 ± 0.04 nm; 5-FU/PFH: 0.98 ± 0.03 nm), supporting molecular dissolution of both payloads in PFH without detectable aggregation or precipitation.

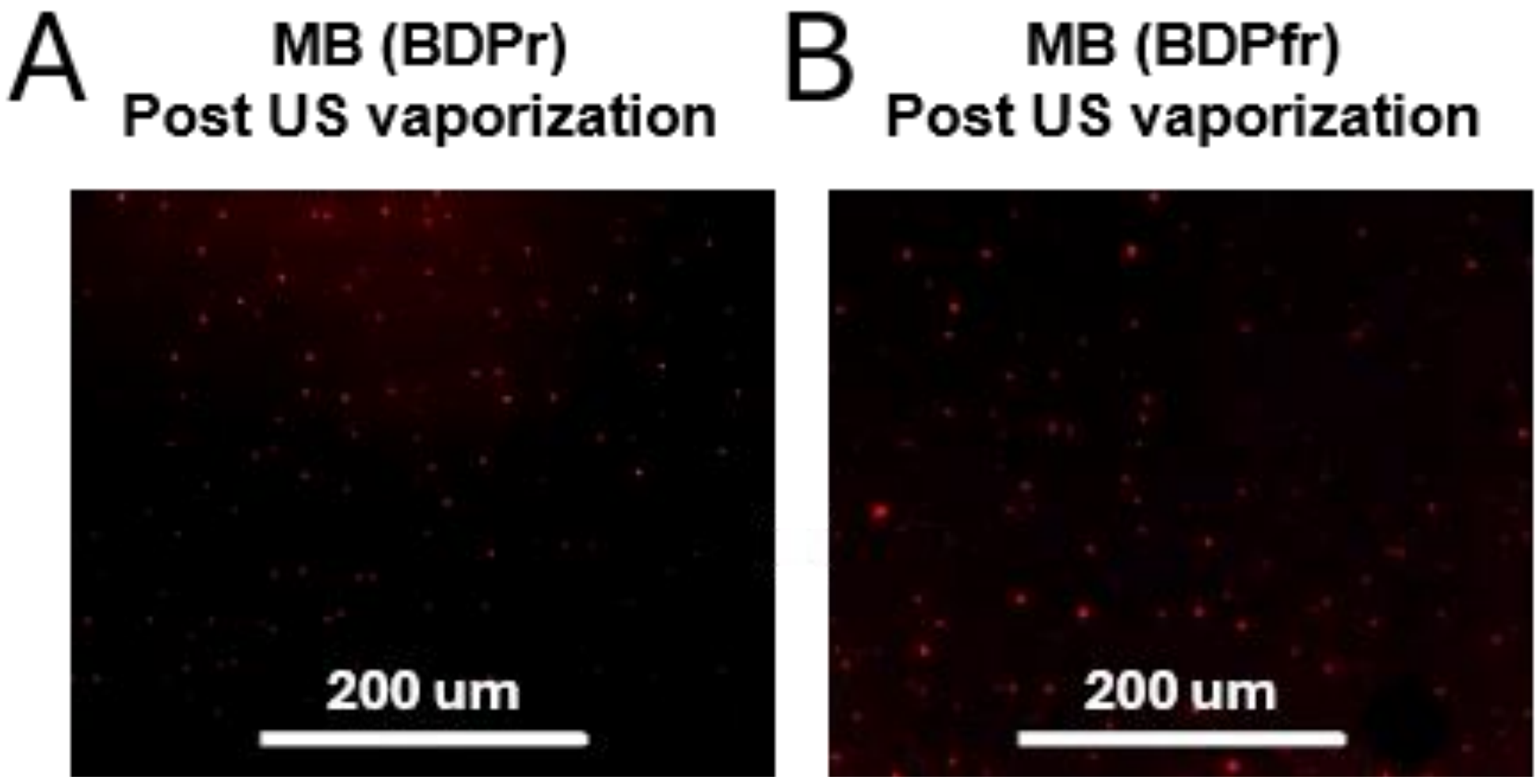

Figure S8. Fluorescence microscopy of BDP-loaded ND following ADV. Scale bar: 200 µm. (A) BDPr ND. (B) BDPfr ND.

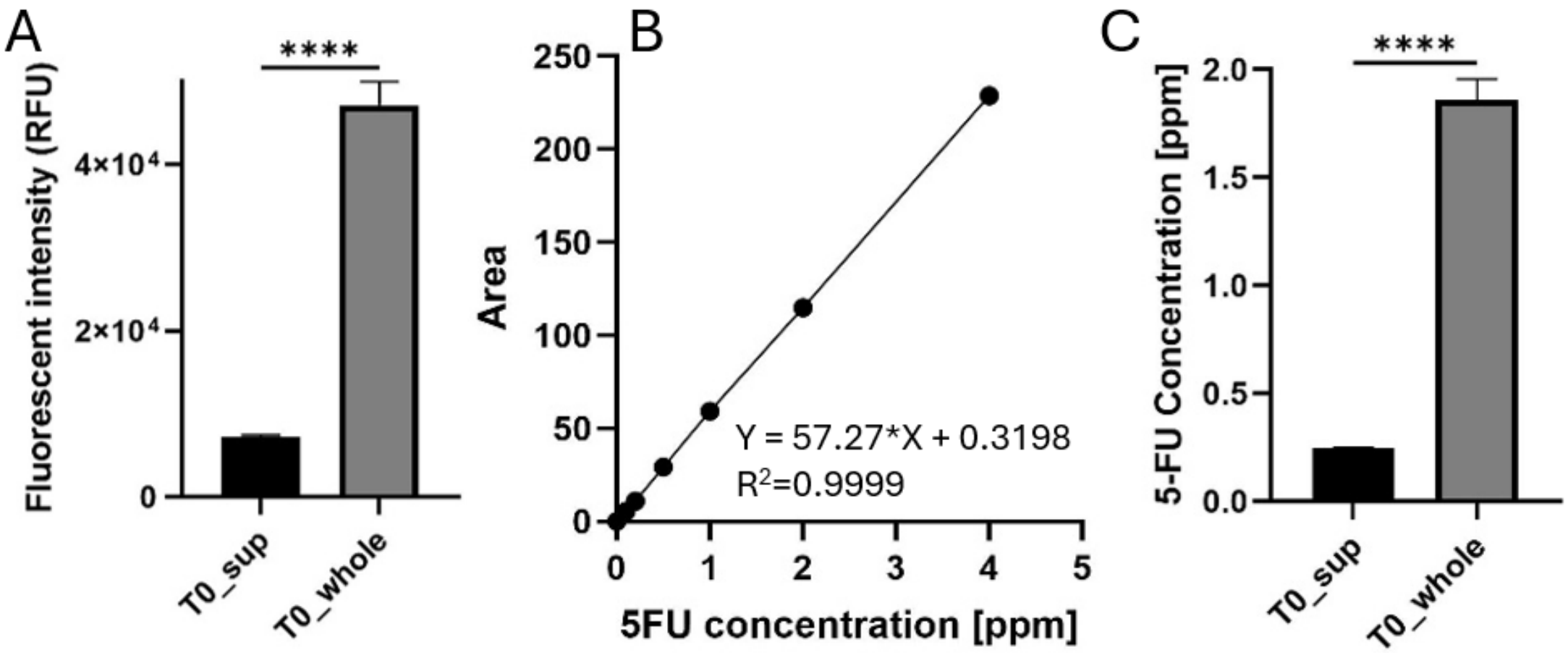

Figure S9. Characterization of BDPg and 5-FU and encapsulation efficiency. (A) Encapsulation efficiency of BDPg, determined by comparing the fluorescence intensity of the supernatant versus the whole formulation (after acetonitrile disruption) at T=0, measured using a plate reader. (B) Calibration curve of 5-FU standard solutions (0, 0.1, 0.2, 0.5, 1, 2, and 4 ppm) obtained via HPLC. The peak area was plotted against concentration to determine the linear regression equation (Y = 57.27*X + 0.3198) and correlation coefficient (R^2=0.9999) for quantification of free 5-FU. (C) Encapsulation efficiency of 5-FU, determined by comparing the 5-FU concentration in the supernatant (unencapsulated/free drug) versus the whole formulation (total drug after acetonitrile disruption) at T=0, measured by HPLC. Data are presented as mean ± SD of n=3 replicates. Statistical comparisons between supernatant and whole samples were performed using an unpaired Student's t-test. ****$p < 0.0001$.

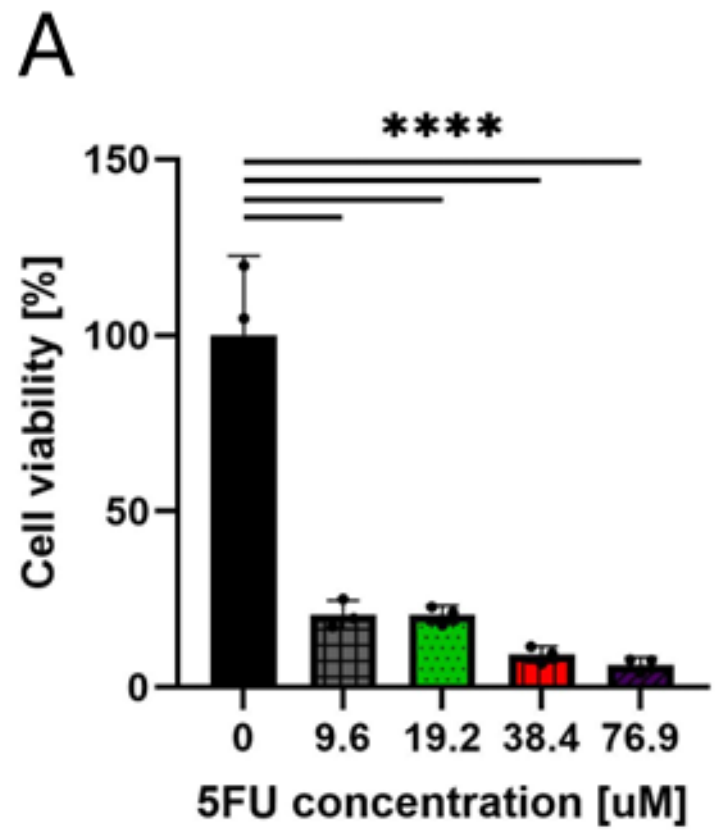

Figure S10. Cytotoxic effect of free 5-fluorouracil (5-FU) on 4T1 breast cancer cells.
(A) Cell viability of 4T1 cells after 48-hour exposure to increasing concentrations of free 5-fluorouracil (0 μM, 9.6 μM, 19.2 μM, 38.4 μM, and 76.9 μM). A strong dose-dependent cytotoxic response was observed, with viability decreasing from 100% in the non-treated control to below 20% at 9.6 and 19.2 μM and under 10% at 38.4 and 76.9 μM. Data represent mean ± SD (n = 3). Statistical significance was determined by one-way ANOVA with Tukey's post-hoc test (****p < 0.0001).

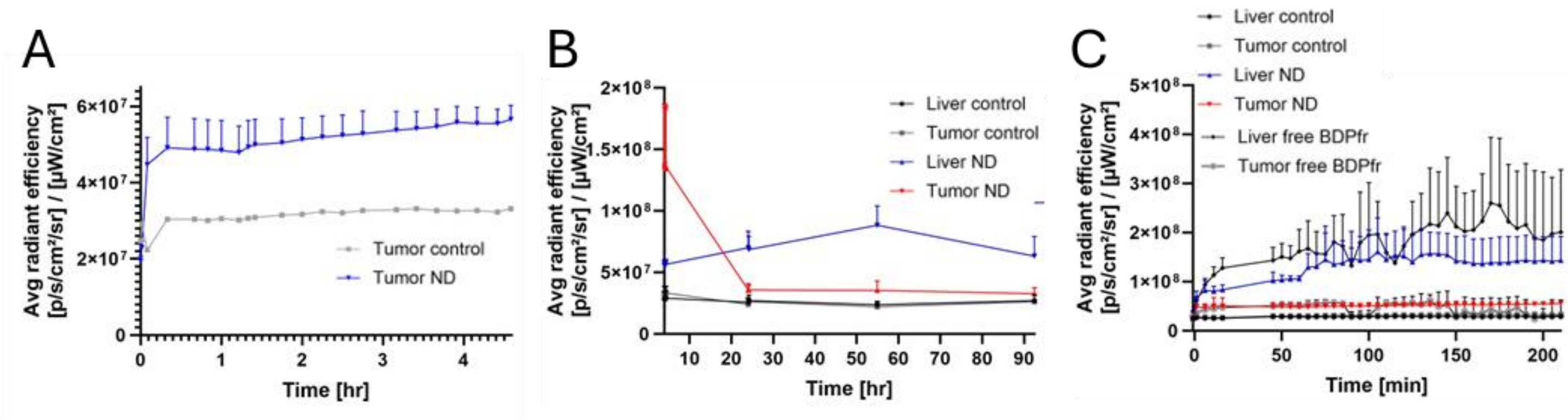

Figure S11. In vivo biodistribution of BDPfr ND. (A) Tumor fluorescence over 4.5 h post-injection, showing rapid ND accumulation and retention. (B) Tumor and liver fluorescence over 90 h post-injection. Liver fluorescence decreased to near-control levels by 24 h, whereas tumor fluorescence increased up to 55 h and remained above control levels at 90 h. (C) Tumor and liver fluorescence kinetics during the first 220 min post-injection, showing early biodistribution of BDPfr-labeled NDs.

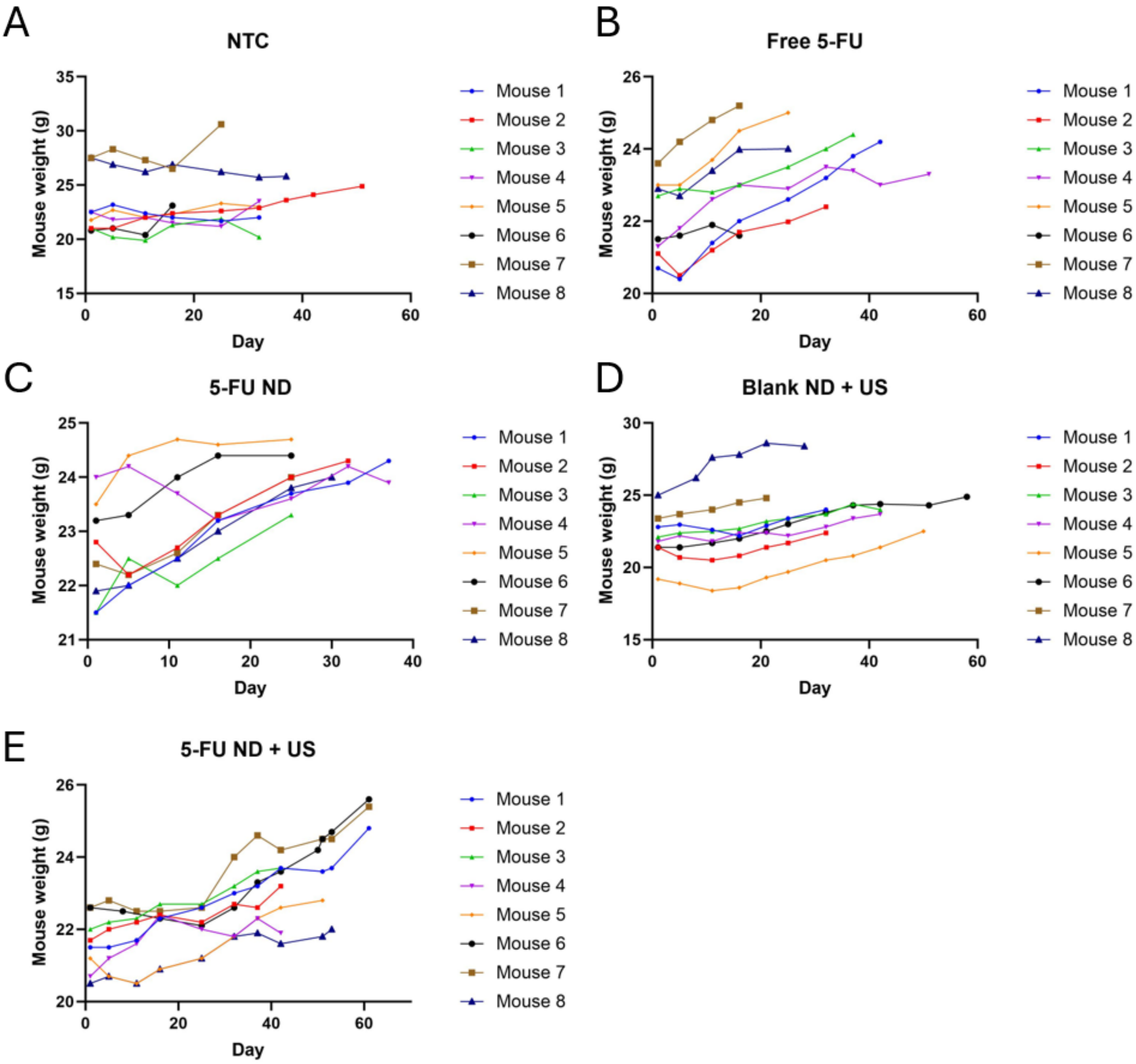

Figure S12. Body weight changes in mice during the survival study. (A-E) Individual body weight for each mouse in the respective treatment groups: (A) NTC, (B) Free 5-FU, (C) 5-FU ND, (D) Blank ND + US, and (E) 5-FU ND + US. No animal exhibited a weight loss exceeding 20% of their baseline weight, confirming that the US-triggered mechanotherapy and localized drug release protocols fell strictly within approved institutional animal care guidelines. The number of mice per group was n=8 at the start of the experiment.
